# Landscape changes elevate the risk of avian influenza virus diversification and emergence in the East Asian–Australasian Flyway

**DOI:** 10.1073/pnas.2503427122

**Published:** 2025-08-18

**Authors:** Shenglai Yin, Chenchen Zhang, Claire S. Teitelbaum, Yali Si, Geli Zhang, Xinxin Wang, Dehua Mao, Zheng Y.X. Huang, Willem Frederik de Boer, John Takekawa, Diann J. Prosser, Xiangming Xiao

**Affiliations:** ^a^School of Biological Sciences, University of Oklahoma, Norman, OK 73019; ^b^U.S. Geological Survey, Eastern Ecological Science Center, Laurel, MD 20708; ^c^U.S. Geological Survey, Georgia Cooperative Fish and Wildlife Research Unit, Warnell School of Forestry and Natural Resources, University of Georgia, Athens, GA 30602; ^d^Department of Environmental Biology, Institute of Environmental Sciences, Leiden University, Leiden 2333 CC, The Netherlands; ^e^College of Land Science and Technology, China Agricultural University, Beijing 100193, China; ^f^National Observations and Research Station for Wetland Ecosystems of the Yangtze Estuary, School of Life Sciences, Fudan University, Shanghai 200438, China; ^g^Key Laboratory of Wetland Ecology and Environment, Northeast Institute of Geography and Agroecology, Chinese Academy of Sciences, Changchun, Jilin 130102, China; ^h^Department of Zoology, School of Life Sciences, Nanjing Forestry University, Nanjing 210037, China; ^i^Wildlife Ecology and Conservation Group, Wageningen University, Wageningen 6708 PB, The Netherlands; ^j^Suisun Resource Conservation District, Suisun City, CA 94585

**Keywords:** waterfowl migration, AIV, agent-based model, habitat availability, mechanistic model

## Abstract

Highly pathogenic avian influenza viruses (HPAIV) persistently threaten wild waterfowl, domestic poultry, and public health. The East Asian–Australasian Flyway plays a crucial role in HPAIV dynamics due to its large populations of migratory waterfowl and poultry. Over recent decades, this flyway has undergone substantial landscape changes, including both losses and gains of waterfowl habitats. These changes can affect waterfowl distributions, increase contact with poultry, and consequently alter ecological conditions that favor avian influenza virus (AIV) evolution. However, limited research has assessed these likely impacts. Here, we integrated empirical data and an individual-based model to simulate AIV transmission in migratory waterfowl and domestic poultry, including wild-to-poultry spillover and reassortment dynamics in poultry, across landscapes representing the years 2000 and 2015. We used the reassortment incidence as a proxy for ecological and transmission conditions that support viral diversification and the emergence of novel subtypes. Our simulations show that landscape change reshaped the waterfowl distribution, facilitated bird aggregation at improved habitats, increased coinfection, and raised reassortment rate by 1,593%, indicating a substantially higher potential for viral diversification and emergence. Model-generated risk maps show expanded and increased reassortment risk in southeastern China, the Yellow River Basin, and northeastern China. These findings suggest the importance of landscape change as a driver of potential AIV diversification and subtype emergence. This underscores the need for interdisciplinary approaches that integrate landscape dynamics, host movement, and viral evolution to better assess and mitigate future risk.

## Significance

Highly pathogenic avian influenza virus (HPAIV) threatens wildlife, agriculture, and humans. Along the East Asian–Australasian Flyway, a major waterfowl migration corridor and HPAIV hot spot, landscape changes are altering migratory bird distributions and increasing opportunities for wild–poultry interactions. By integrating empirical data into an individual-based model, we show that landscape change between 2000 and 2015 reshaped waterfowl migration, substantially increased wild-poultry spillover, and avian influenza virus (AIV) reassortment in poultry, our proxy for potential AIV diversification and emergence of novel subtypes. Risk regions expanded across southeastern China, the Yellow River basin, and northeastern China. These findings highlight the importance of landscape changes in potentially elevating AIV diversification and emergence, and the landscape dynamics should be integrated into future studies.

Since its first detection in domestic poultry in 1996 (1), highly pathogenic avian influenza virus (HPAIV) A/H5N1 has caused widespread outbreaks, affecting poultry industries and wild bird populations (2). Over time, HPAIV H5 subtypes have evolved, with clade 2.3.4.4b variants, particularly the subtypes of H5N8 and more recently emerged H5N1, spreading across new geographic regions (3, 4) and hosts (5), including dairy cattle (6), which has increased concerns for public health, agriculture, and wildlife. HPAI H5N8 clade 2.3.4.4b was circulating in wild bird populations prior to May 2020 (7, 8), whereas through genetic reassortment with other low pathogenic avian influenza viruses (LPAIV), these H5N8 strains led to the emergence of a new H5N1 subtype, which likely enhanced the virus’s adaptability and facilitated its rapid spread across various host groups (9).

Genetic reassortment, the exchange of gene segments between coinfecting subtypes within a host (10), is an important mechanism for increasing the diversity of viral genotypes (11) and subsequently gives rise to new subtypes (10, 12). While reassortment alone does not guarantee high pathogenicity (10, 13), it can result in highly pathogenic strains when one of the coinfecting viruses is already an HPAIV (10), as exemplified by the emergence of H5N1 clade 2.3.4.4b (8, 9). In most cases, however, high pathogenicity in avian influenza virus (AIV) arises from specific mutations, such as polybasic cleavage site insertions in the HA gene (10). Nonetheless, reassortment plays a crucial role in contributing to the genetic diversity of AIVs, some of which may later acquire mutations for high pathogenicity under favorable ecological conditions (10).

The first HPAIV isolation from wild waterfowl occurred in the East Asian–Australasian Flyway (EAAF) in 2002/2003 (14), and the HPAIV infection in wild waterfowl was initially regarded as a spillover from domestic poultry (14). Since then, HPAIV has persisted in the flyway, associated with large populations of migratory waterfowl and domestic poultry (15). Each year, millions of waterfowl migrate from northern breeding grounds in Siberia and Mongolia to wintering grounds in the Yangtze River Basin in southeastern China. Seasonal migration, especially in the fall, facilitates the long-distance spread of the virus (16, 17), increases local viral diversity and coinfection risk, as well as the risk of novel viral emergence (18, 19).

Waterfowl distribution during migration depends on landscape features, particularly the availability of surface water, wetlands, and rice paddies, for roosting and foraging (20–22). However, the EAAF landscape has changed considerably in recent decades. For example, surface water has substantially decreased in northern China, while expanding in the south (23). Wetland area has generally declined in eastern China, with some local increases in the northeast and central-southern regions (24). In addition, rice paddies, which serve as high-quality foraging habitats for both wild waterfowl and domestic poultry, have declined in southern China but expanded in the north in recent years (25, 26). These landscape alterations directly affect waterfowl distribution across the flyway, either aggregating birds in fewer remaining suitable areas (21, 27) or attracting more birds to new suitable areas (28).

These landscape-driven shifts in waterfowl distribution influence AIV dynamics in two ways. First, aggregation due to habitat loss can increase local bird density and facilitate AIV transmission (29). Second, increased use of habitats shared with poultry (e.g., rice fields) can promote wild–domestic contacts and, consequently, spillover transmission (28). Both ways can increase the opportunities for coinfection and reassortment that can potentially lead to an increase in AIV viral diversity and the emergence of novel subtypes. However, to date, no study has comprehensively assessed how landscape changes affect the risks through their influence on bird migration.

Here, we evaluate how landscape changes in the EAAF from 2000 to 2015 have affected migratory waterfowl distribution and the potential risks of viral diversity and novel subtype emergence. We combined telemetry tracking data from a migratory waterfowl host, Greater White-fronted goose (*Anser albifrons*, GWFG), eBird data, landscape data, and poultry distribution data to develop an individual-based model (IBM) that simulates waterfowl movements and cross-species transmission at the wild bird–poultry interface. The model combines a migratory flow network model (30), where nodes represent sites and edges represent potential movement paths (29), with compartment models (i.e., Susceptible–Infected–Recovered). Bird movements are determined by habitat availability and distance, and the model simulates infection dynamics within wild and poultry populations, spillovers from wild birds to poultry, and reassortments in poultry. By comparing simulations between 2000 and 2015, we assessed how landscape change influences these risks through altering bird migration, using reassortment incidence as a proxy.

## Results and Discussion

### Impacts of Landscape Changes on Wild Bird Migration Dynamics.

**Fig. 1.**
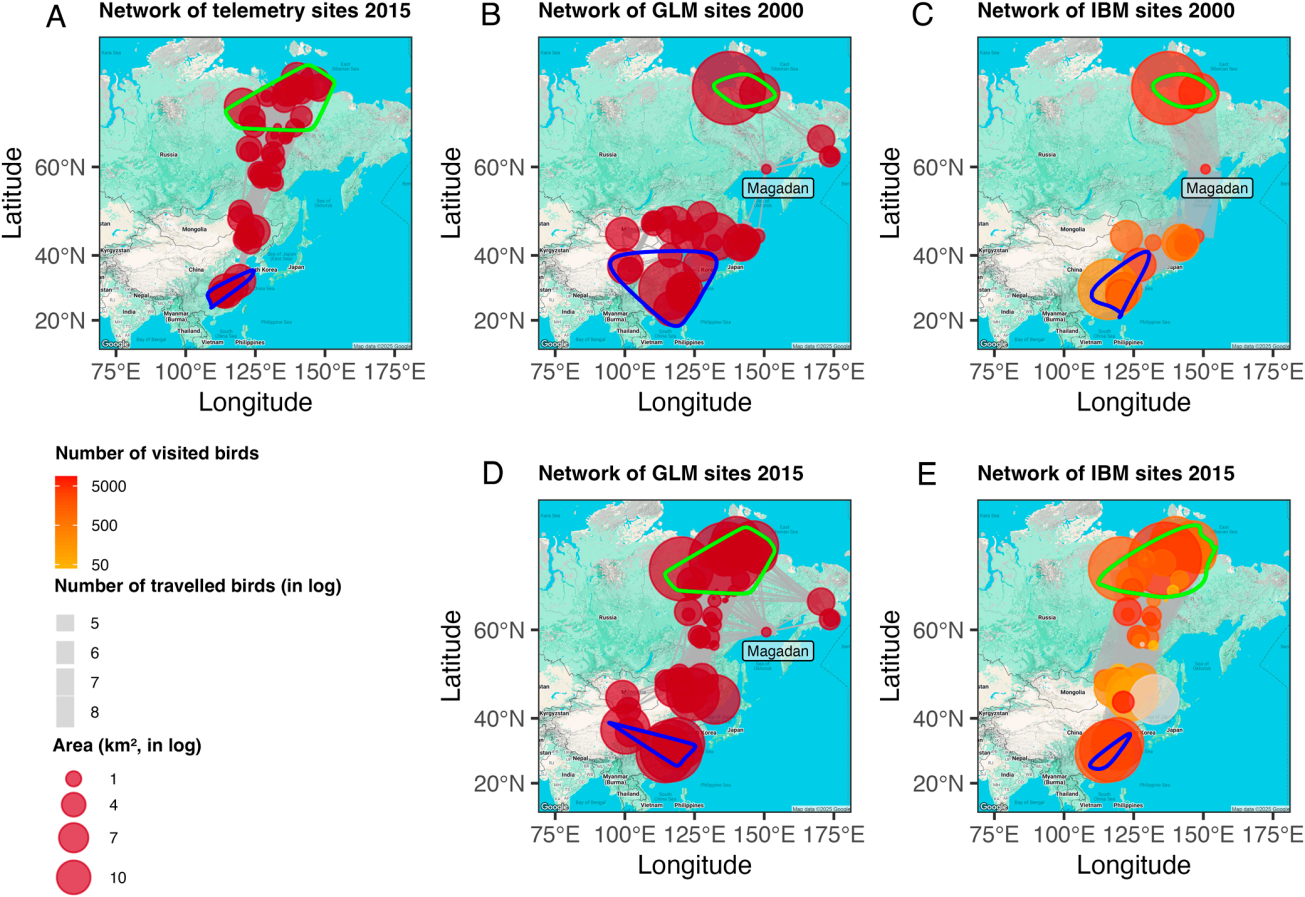
Migration networks generated from telemetry tracking, GLM predictions, and IBM simulations for 2000 and 2015 scenarios. (*A*) Network of sites from dBBMM using 2015 telemetry tracking data; (*B*) Network of sites predicted by GLM for 2000; (*C*) Network based on sites simulated by IBM for 2000; (*D*) Network of sites predicted by GLM for 2015; (*E*) Network based on sites simulated by IBM for 2015. Links in networks *A*, *B*, and *D* use migration step length, whereas links in networks *C* and *E* use simulated IBM movement trajectories. Green and blue contours show breeding and wintering ground ranges, respectively. In networks *C* and *E*, node color indicates number of visited birds, and link width indicates number of traveling birds.

Telemetry tracking data revealed 50 sites used by GWFG between 2014 and 2016, including 11 breeding, 7 wintering, and 32 stopover sites (Fig. 1*A* and Dataset S1). Based on the tracking data and environmental variables, we built a generalized linear model (GLM) to predict suitable sites for 2000 and 2015. The number of predicted stopover sites in Russia increased between 2000 and 2015, decreasing the strong reliance on the Magadan site (Fig. 1 *B* and *D*). Meanwhile, breeding and wintering sites showed contrasting trends. The number of breeding sites increased from 2 to 14 (Fig. 1 *B* and *D*), with a 23.3% increase in habitat availability (i.e., sum of wetland and rice paddy area). Although wintering sites decreased from 13 to 5, habitat availability increased by 39.7%, driven by a 398.2% expansion in wetland area, offsetting a 15.9% decline in rice paddy area (Datasets S2 and S3). IBM simulations showed that improved connectivity and habitat in 2015 shifted birds from heavy reliance on the poorly connected Magadan site to a broader network across Russia and the borders of Mongolia and northeast China (Fig. 1 *C* and *E* and *SI Appendix*, Table S1).

The changes in site use reflect broader habitat changes across the EAAF. Before 2000, widespread habitat degradation and loss restricted waterbird populations and distributions (22, 31). For example, an Oriental white stork (*Ciconia boyciana*) population had been restricted to relying on a single stopover site to finish its migration around 2000 (32). Similarly, in our 2000 scenario, GWFG had limited stopovers in Russia, increasing their dependence on a few key sites. However, by 2015, habitat availability had increased in Russia and northeast China (see also ref. 33), with more sites at higher latitudes and areas with increased surface water (Fig. 1 *C* and *E* and *SI Appendix*, Table S2), likely due to climate change-related increases in surface water (34, 35) and/or from agricultural land abandonment in Russia (36). These changes allowed shorter, more frequent stopovers, reducing dependence on Magadan. Meanwhile, substantial habitat loss persisted in Japan and the Korean Peninsula (Fig. 1 *D* and *E*), consistent with long-term observations (33, 37, 38). Despite fewer wintering sites, total wintering area remained stable (9,603 km^2^ in 2,000 vs. 9,520 km^2^ in 2015; Datasets S2 and S3)(24, 39), with birds in the 2015 scenario concentrating in fewer but larger sites, primarily in the Yangtze River Basin.

**Fig. 2.**
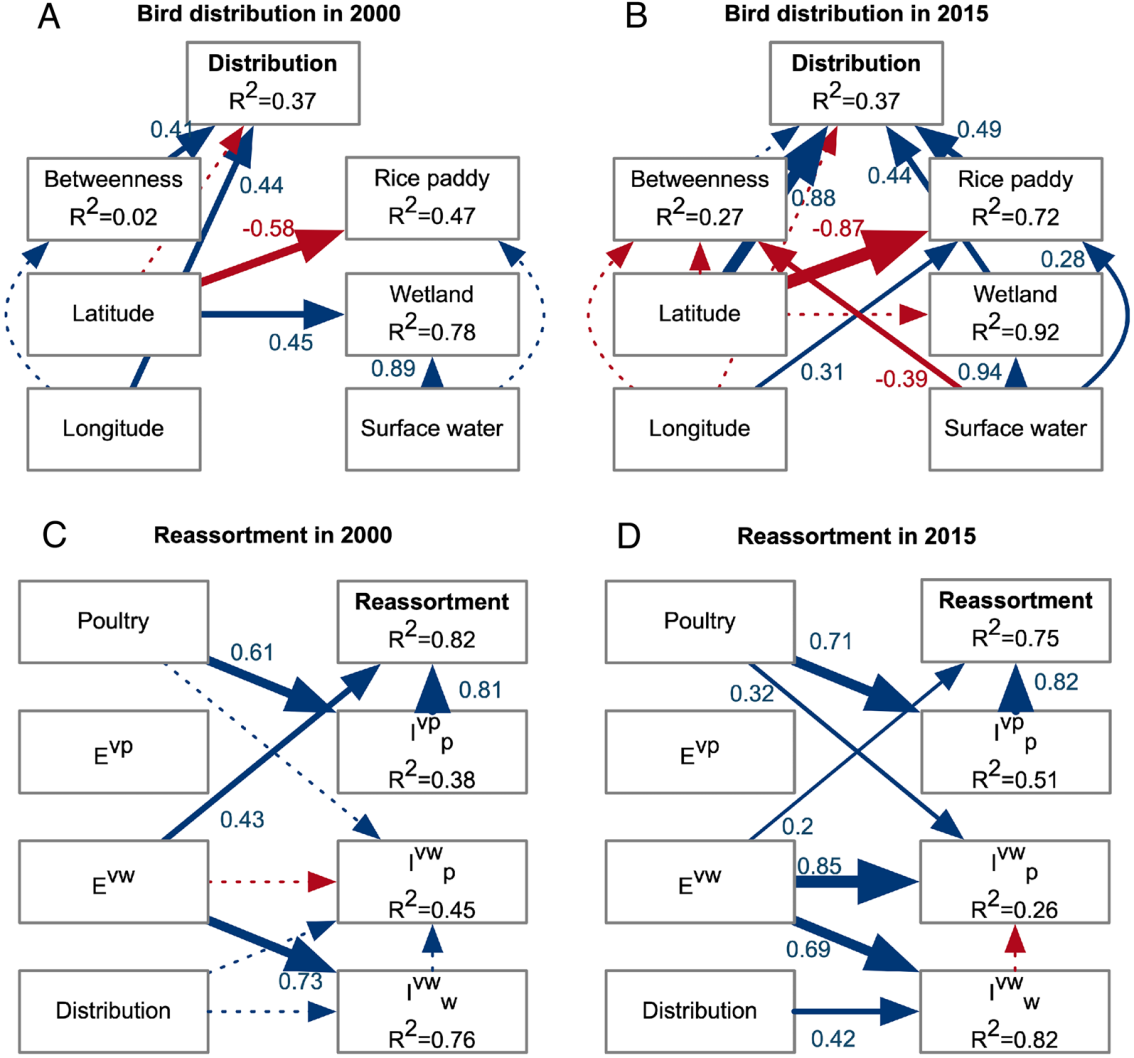
Results from piecewiseSEM analysis based on IBM simulation outputs. (*A*) drivers of bird distribution in 2000 and (*B*) 2015; (*C*) drivers of reassortment in 2000 and (*D*) 2015. In panels *A* and *B*, Distribution represents cumulative number of visited GWFG, Rice paddy, Wetland, and Surface water represent the areas of the landscape type. In panels *C* and *D*, Reassortment is accumulated incidence, Poultry indicates poultry abundance, E and I represent environmental viral load and infected birds, respectively, with subscripts w and *p* for wild and poultry birds, and superscripts vw and vp for LPAIV_w_ and LPAIV_p_. Arrows indicate causal paths: Solid lines denote significant effects (*P* ≤ 0.05), dashed lines nonsignificant, blue and red indicate positive and negative effects. The size of each arrow corresponds to the effect size, which is also annotated next to the arrows.

Habitat loss can force birds to concentrate in fewer sites, restricting their spatial distribution (21, 40), while increased availability allows dispersal (29, 33) and colonization of new sites (41, 42). Our piecewise structural equation modeling (piecewiseSEM) results align with these findings, showing that in 2000, bird distributions were mainly constrained by low network connectivity (Fig. 2*A*), relying on a few connecting sites (Fig. 1 *B* and *C*). In 2015, wetland and rice paddy became more important in shaping GWFG distribution (Fig. 2*B*), particularly in northeast China, where expanding rice cultivation attracted birds (Fig. 1 *D* and *E* and *SI Appendix*, Fig. S4 *A* and *B*) (25, 26, 28). The increased importance of habitat in 2015 compared to 2000 (Fig. 2 *A* and *B*), combined with major landscape changes over time, highlights the importance of landscape change in reshaping bird distribution along the flyway (33).

### Simulated Changes in Transmission within and Across Species.

**Fig. 3.**
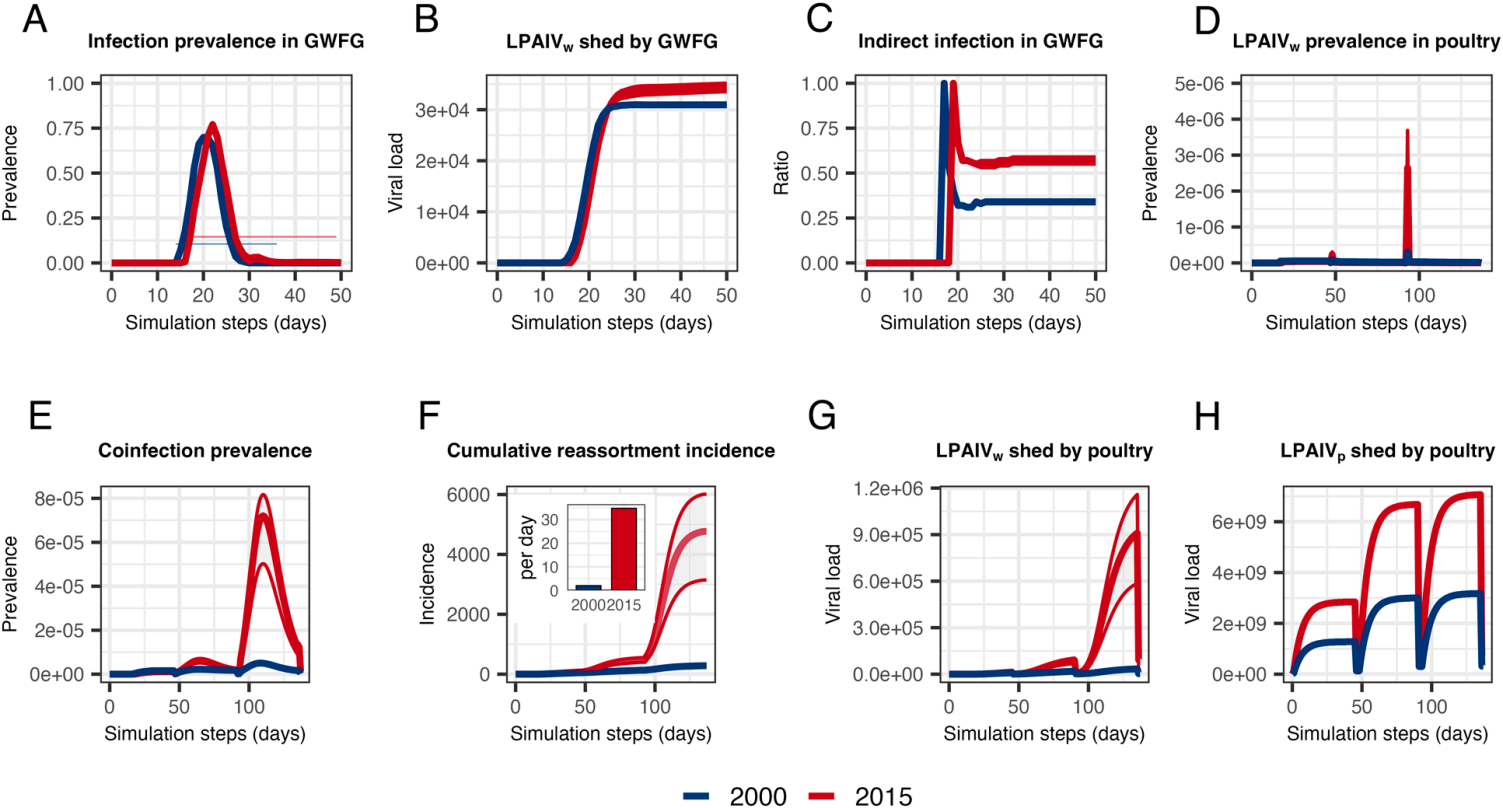
Epidemiological dynamics in GWFG and poultry as simulated in the IBM. (*A*) Infection prevalence of LPAIV_w_ in GWFG; (*B*) Environmental viral load LPAIV_w_ shed by GWFG; (*C*) Contribution of environmental indirect transmission in GWFG; (*D*) Infection prevalence of LPAIV_w_ in poultry; (*E*) Coinfection prevalence in poultry; (*F*) Cumulative reassortment incidence, with inset showing reassortment rate; (*G*) Environmental viral load LPAIV_w_ shed by poultry; and (*H*) Environmental viral load LPAIV_p_. Blue and red indicate the years 2000 and 2015, respectively, and ribbons indicate the SD of the mean, and horizontal segments in (*A*) indicate the migration phases.

Increased breeding sites from 2000 to 2015 prolonged the period over which GWFG departed for fall migration (*SI Appendix*, Fig. S5*A*), delaying virus exposure and transmission at stopovers. This resulted in higher infection prevalence during the declining phase after the peak (Fig. 3*A*), particularly during stopover and winter arrival in 2015 (Fig. 3*A* and *SI Appendix*, Fig. S5 *B* and *C*). The elevated infection prevalence increased the risk of viral spread from stopover to wintering sites and increased cross-species transmission by boosting environmental viral loads at wintering sites (Fig. 3*B*). LPAIV_w_ load was the leading contributor to infections in wild birds in both years, causing 57% of infections in 2015, compared to 34% in 2000 (Fig. 3*C*), highlighting the role of environmental transmission (43–45).

For poultry hosts, LPAIV_p_ prevalence remained comparable between the years and was regulated by disinfection after periodic trading events (*SI Appendix*, Fig. S6). However, LPAIV_w_ prevalence, coinfection, and cumulative reassortment incidence were substantially higher in 2015, with reassortment rate peaks 15.9 times higher than in 2000 (Fig. 3 *D*–*F*). For both LPAIV_p_ and LPAIV_w_, increased viral loads shed by poultry (Fig. 3 *G* and *H*) followed infection dynamics (Fig. 3 *D*–*F*), indicating amplification of cross-species transmission.

PiecewiseSEM identified environmental LPAIV_w_ load (E^vw^) and the poultry LPAIV_p_ prevalence (I_p_^vp^) as the main drivers of reassortment incidence (Reassortment) in both years (Fig. 2 *C* and *D*). This is expected, as reassortment occurs only when wild- and poultry-origin LPAIVs coinfect the same host in the model. Although we did not find a statistical association between the cumulative number of visited birds (Distribution) and reassortment incidence, given that LPAIV_w_ load (E^vw^) originates from infected wild birds and significantly influences reassortment, we infer that the cumulative number of birds has a fundamental impact on reassortment. In addition, the role of the LPAIV_w_ load (E^vw^) also indicates that cross-species transmission drives the disease dynamics that lead to reassortment. Sensitivity analysis supports this by showing that the cross-species transmission rate disproportionally influences reassortment, especially in the 2015 scenario (*SI Appendix*, Fig. S7).

Poultry LPAIV_p_ prevalence (I_p_^vp^) was positively influenced by poultry population size (Poultry). The effect was larger in 2015 (β=0.71 vs. 0.61 in 2000, Fig. 2 *C* and *D*), suggesting that altered landscape conditions in 2015 enhanced the role of poultry in sustaining LPAIV_p_ circulation. These findings emphasize the need for biosecurity practices, such as separating poultry from wild birds (46) and controlling viral circulation within poultry through measures like disinfection and flock management (47, 48), to minimize the risk of spillover and novel subtype emergence.

### Simulated Changes in Spatial Risk of AIV Reassortment in 2000 and 2015.

**Fig. 4.**
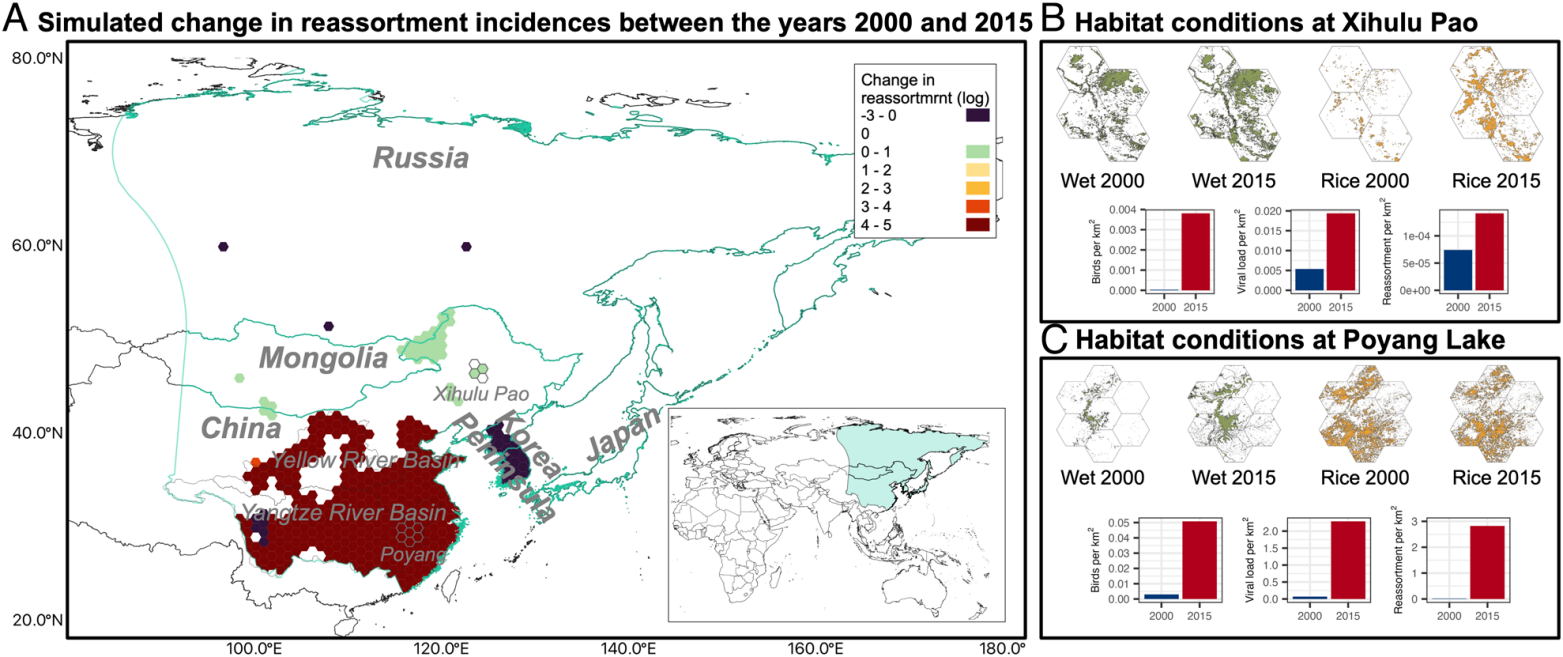
Simulated changes in reassortment incidences and associated habitat conditions between 2000 and 2015. (*A*) Map showing change in reassortment incidence (log-transformed) between 2000 and 2015, and the inset map provides geographic context showing the study region; (*B*) Habitat characteristics and simulation outcomes for Xihulu Pao, including maps of wetland (Wet) and rice paddy (Rice) distribution in 2000 and 2015, and bar charts comparing simulated number of visited geese, environmental viral load of LPAIV_w_, and reassortment incidence per km^2^ between years; (*C*) Corresponding habitat characteristics and simulation outcomes for the Poyang Lake. The light blue shaded area in (*A*) represents the study region boundary.

Our simulations suggest that reassortment risk increased in both magnitude and spatial extent between 2000 and 2015, especially in northeastern China, the borders with Mongolia and Russia, and from the Yangtze to Yellow River Basin (Fig. 4*A*). These increases are associated with improved habitat conditions that enhanced site attractiveness and wild bird population. For example, both wetland and rice paddy expanded at Xihulu Pao in northeastern China (Fig. 4*B*), and wetland area increased, despite a minor decrease in rice paddy, at Poyang Lake in southeastern China (Fig. 4*C* and *SI Appendix*, Fig. S4 *A* and *B*) (23, 24, 26, 39). These landscape changes made these sites more attractive to wild birds in our model (see Eqs. **1** and **2** in the *Materials and Methods*), leading to more visiting birds, and consequently, higher viral loads and increased reassortment rates (Fig. 4 *B* and *C*). These outcomes support previous studies suggesting that increased rice paddy attracts more migratory waterfowl, and when domestic poultry also use these areas, the co-occurrence can increase the AIV spillover risk (28, 49).

Additionally, our finding of elevated reassortment risk also aligns with historical patterns of HPAIV reassortment events and empirical observations of bird migration. Specifically, no HPAIV reassortments were reported before 1995, but 45 events occurred during 1996–2005 and 82 during 2006–2015 in East Asia (10), mirroring the landscape-driven increase in reassortment risk observed in our simulations. Moreover, since 2000, waterbirds from Siberia and Mongolia have increasingly used the Yellow River Basin as a stopover, year-round, or overwintering site (50, 51), facilitating the introduction and spread of H5N8 clade 2.3.4.4b in the region (50, 52).

### Limitations and Future Modeling Efforts.

This study focused on GWFG due to the availability of high-resolution telemetry tracking data and well-characterized habitat preference. While GWFG uses croplands and interacts with poultry (53), our GWFG-focused model likely underestimates overall transmission, which is dominated by other key hosts, especially dabbling ducks (54). AIV dynamics are complex, involving multiple species with differing habitat needs, movement patterns (33), and viral shedding characteristics that can significantly alter spillover risk (55). Gulls, for example, use different habitats than geese but play an increasingly important role in recent HPAIV H5 clade 2.3.4.4b transmission (56). These interspecific differences can influence how species respond to landscape changes and their roles in AIV transmission. Our single-species model provides a proof-of-principle that landscape change can influence emergence risk via host movement and poultry contact and should be interpreted as a baseline that illustrates these mechanisms, rather than a comprehensive or quantitative risk assessment.

Wild bird migration is influenced by factors beyond habitat availability, including weather conditions such as temperature (57, 58). Temperature generally influences migration timing, especially for departures from breeding and wintering sites (53, 59), often in interaction with habitat conditions such as vegetation growth. This study focused on habitat availability for simplicity, but future research aiming to comprehensively model waterfowl behavior, distribution, and associated AIV risk may consider incorporating weather conditions and other migration drivers. Integrating weather variables would also enable simulations of climate change effects on AIV dynamics, which have important impacts on the emergence of novel subtypes, especially for the emergence of HPAIV (60).

While we aimed to assess AIV diversification and the potential emergence of novel subtypes driven by landscape changes, we did not explicitly model the complete molecular and evolutionary processes, such as natural selection and mutation, but instead used reassortment incidence in poultry as a proxy. Thus, our simulation outputs should be interpreted as indicators of ecological and transmission conditions that heightened the potential for the risks, rather than as quantitative predictions. We did not include the mutation mechanisms required to generate high pathogenicity, but given the widespread circulation and significant impact of H5 2.3.4.4b, it is important to incorporate the mutation dynamics for modeling HPAIV emergence risk (9). However, mutation rates remain insufficiently quantified across most host taxa, including GWFG (61), which constrained our ability to assess the HPAIV emergence risk in this study. Future modeling studies that incorporate more comprehensive evolutionary mechanisms, including natural selection and host-specific mutation probabilities where such data are available (61), will improve the prediction of viral diversification, as well as the transition to high pathogenicity under macroecological drivers such as landscape and climatic change.

We used a spatial proximity approach to define sites, simplified the heterogeneous landscape by merging those within a 15 km buffer zone (2 × 7.4 km of foraging distance) into a single site, and summarized them by geographic centroids (27, 29). This approach resulted in some unrealistically large sites, particularly in Siberia and the Yangtze River Basin. For example, birds in eastern Siberia are unlikely to interact with those in the west due to the vast distances and low activity during the breeding season (62). This simplification could have affected GWFG’s spatial spread and local density, thereby influencing transmission efficiency. We partially addressed this by testing LPAIV_w_ exposure at different migration phases (i.e., breeding, migration, and overwintering). Since both density and exposure timing affect transmission, early exposure allows the pathogens more time to spread within populations, while later exposure limits their spread across the network. This approach partially accounts for density-driven differences in infection dynamics. Our consistent finding of a higher reassortment rate in 2015 across scenarios suggests this simplification is unlikely to affect our qualitative results.

## Summary

Our simulations demonstrate that landscape changes between 2000 and 2015 alone can reshape migratory waterfowl distribution in EAAF, increasing interactions with poultry and elevating reassortment incidences, a proxy for risks of viral diversification and subtype emergence. By integrating ecological and epidemiological modeling, our findings extend previous phylogenetic and virological studies on the mechanisms driving the risks (9, 63, 64), highlighting the critical role of environmental changes in AIV dynamics. As landscape changes continue to reshape the EAAF and other migratory flyways, an interdisciplinary approach combining ecology, molecular biology, computational modeling, and macroecological drivers will be essential for predicting AIV dynamics and identifying high-risk zones.

## Materials and Methods

Our study area is located in the EAAF. Since we simulated the migration of GWFG, we extracted landscape and environmental variables within the species’ range in the flyway (33). The range, which has been described in previous studies (29, 33), stretches from Siberia, passing through Mongolia, to the middle and lower reaches of the Yangtze River Basin in China and extends to the Korean Peninsula and Japan (Fig. 4*A*).

### Landscape and Environmental Data.

We collected remote sensing-derived data layers for the years 2000 and 2015 to assess changes in GWFG habitats, including surface water, wetlands, and rice paddies. Densities of humans and roads were collected to control for GWFG observation bias in the eBird dataset, and density of poultry (i.e., domestic duck) was used to characterize the poultry population size at each GWFG site.

We converted surface water data into vector format and built buffer zones of 7.4 km (i.e., foraging distance for GWFG)(65) outward from each surface water boundary and merged any overlapped buffers to treat adjacent surface water bodies as one (27, 29). We calculated the area and perimeter of each merged surface water polygon. We also calculated wetland area and perimeter, rice paddy area, and average densities of humans, roads, and poultry inside each polygon (see detailed environmental data in *SI Appendix*, Table S3).

### eBird and Satellite Telemetry Data.

We used field observation records from eBird between 1995 and 2020 to describe potential sites for GWFG (66). This broader time window compensates for the limitations of eBird data in East Asia. We retained 4,083 GWFG locations (see the raw eBird locations in *SI Appendix*, Fig. S8) after filtering the data according to the eBird data processing procedures (67).

We used satellite telemetry tracking data (late 2014 to 2016) from 79 GWFG individuals to further select suitable sites for the 2015 scenario. Geese were equipped with GPS–GSM (Global Positioning System–Global System for Mobile Communications), solar-powered neckband devices at Poyang Lake (29.1˚N, 116.3˚E) in the winter of 2014/15 to record 12 GPS locations per day for each individual (see raw GPS locations in *SI Appendix*, Fig. S9). Deployment details of the tracking device have been described in previous studies (53, 59).

To include most of the sites the tracked geese used, we segmented each individual’s tracking trajectory by season and year and outlined seasonal sites (i.e., breeding, stopover, and wintering sites) based on the distribution of GPS locations. We first generated a net displacement plot for each individual (*SI Appendix*, Fig. S10) and identified the turning point for seasonal behavior change (i.e., departure for migration or arrival) in interactive HTML files of the net displacement plots (Dataset S4) to separate the seasons in each year. After excluding the segments of spring migration and without a complete migration journey from breeding to wintering sites, we included 257 fall segments for further analysis. Following previous studies (68, 69), we fit a dynamic Brownian Bridge Movement Model (dBBMM) to each segment to calculate a utilization distribution. We used a 10 × 10 km resolution with a window size of 31 GPS locations (i.e., approximately 2 to 3 d) and a margin size of 11 locations, treating 50% cumulative probability contours as sites (31, 68).

### Identification of Suitable Sites with Generalized Linear Model.

We assume dBBMM sites represent precise locations used by the tracked GWFG population, while eBird observations provide broader, opportunistic records that may include sites used by other populations or reflect low-quality or transient use rather than core sites. Thus, to identify a broader range of suitable sites for the tracked population, we combined dBBMM sites and eBird observation sites to compile a GWFG presence and pseudoabsence dataset alongside environmental variables for identifying suitable sites for 2015. Sites were defined as surface water polygons intersecting either dBBMM or eBird locations; those overlapping dBBMM were considered presences, and the rest pseudoabsences. Predictor variables included the area and perimeter of surface water, wetlands, and rice paddies, densities of humans, roads, and domestic ducks, along with geographic coordinates (i.e., longitude and latitude).

Due to the unbalanced data (50 presence vs. 176 pseudoabsence sites), we applied 1,000 replicated bootstrapped sampling and univariate regressions, averaging the results across replicates to identify significant predictors (*SI Appendix*, Table S4), following a previously published method (70). To avoid multicollinearity, we examined pairwise correlations and removed predictors with correlation coefficients greater than 0.7 (*SI Appendix*, Table S5). Variables with *P*-values smaller than 0.05 were retained for the final multiple GLM regression. This final model also used a bootstrapped sampling procedure, and sites with predicted probabilities greater than 0.6 were classified as suitable, following a previous study (33). The model achieved 88.5% overall classification accuracy based on agreement with dBBMM (*SI Appendix*, Table S6). Road density was included in all GLMs to account for sampling bias.

**Fig. 5.**
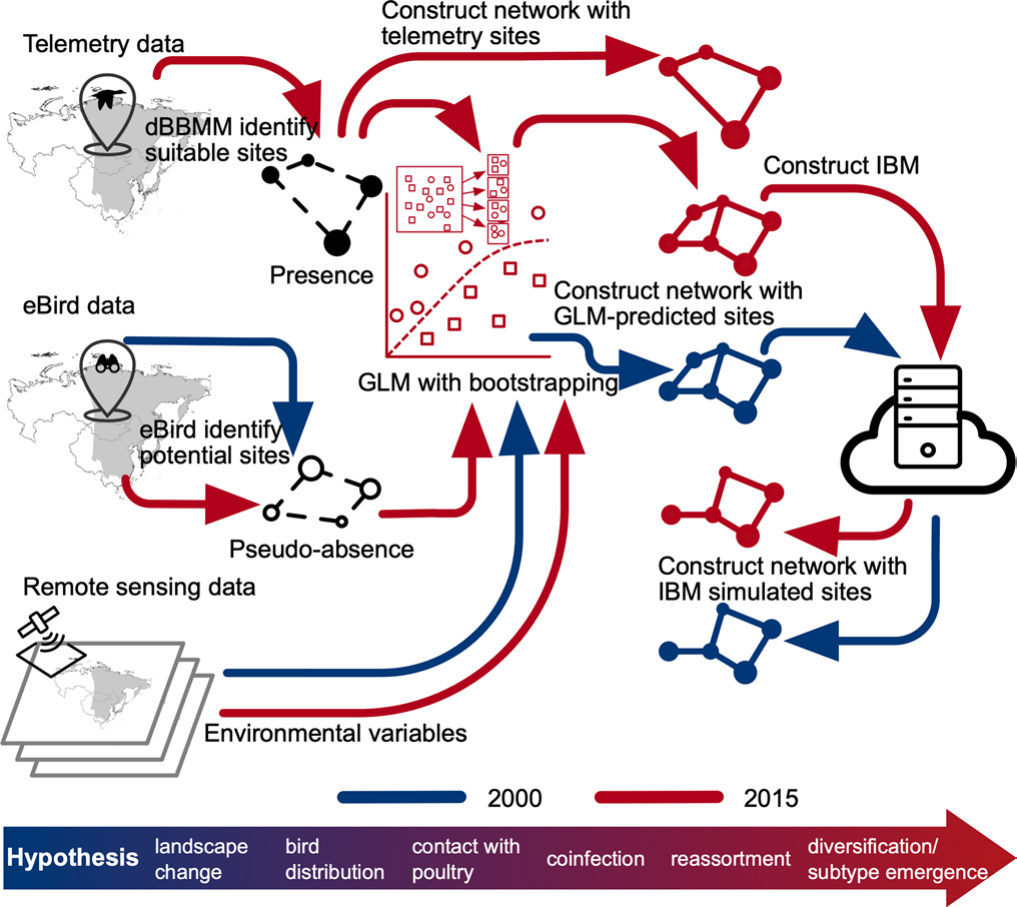
Schematic of the data preparation and processing flow. Red and blue elements in the diagram represent procedures for 2000 and 2015 scenarios, respectively.

Because tracking data were only available for 2014–2016, we applied the 2015 model to environmental conditions in 2000 to backcast site suitability. The potential sites, their associated environmental variables, and the predicted probability in both years are included in Datasets S2 and S3, respectively. A schematic of the data preparation and processing flow is illustrated in Fig. 5.

### Individual-Based Model Construction.

#### Simulating bird migration.

Using the GLM-predicted suitable sites, we constructed fall migration networks for 2000 and 2015, following previous approaches (27, 29). Suitable sites were treated as nodes, connected by directional links (i.e., north to south) if their distance was less than the migration step length (*S_length_*, see parameterization below). We imported these networks into an IBM platform (71) and initialized 10,000 individuals that followed behavioral rules adapted from a prior study (29). Individuals were initially distributed across breeding sites in proportion to the resource availability (i.e., the sum of wetland and rice paddy areas). Each individual was assigned a random initial body mass (*B_mass,t=0_*) drawn from a truncated normal distribution defined by species’ mean body weight (see parameterization below) (29). Birds departed breeding sites when body mass reached a threshold (*T_mass_*), lost body mass during flight, and gained body mass while refueling at stopovers. Stopover duration was regulated by *R_rest_*, the ratio of resting time to total migration time, based on tracking data indicating that GWFG spend 60 to 70% of their migration period resting (72).

The migratory flow network model determined bird trajectories (29, 30), using site-level habitat attractiveness (*A_i,t_*), which followed a logistic growth curve and was proportional to per capita resource availability. We assumed constant resource availability over time during fall migration (72):

[1] $$A_{i,t}=\frac{{\mathrm{Res}}_{\max}}{1+e^{\left(a\times Res_{\mathrm{med}}-k_{i,t}\right)}},$$

where *Res_max_* and *Res_med_* are the maximum and median resource values across sites, *α* is a scaling parameter to control the shape of the function curve, and *k_i,t_* is the per capita resource:

[2] $$\begin{array}{c} k_{i,t}=\left\{\begin{array}{c} \frac{{\mathrm{WetArea}}_{i,t}+{\mathrm{RiceArea}}_{i,t}}{N_{w,i,t}},if\;N_{w,i,t}>0\\ {\mathrm{Wet}}_{i,t}+{\mathrm{Rice}}_{i,t},otherwise \end{array}\right., \end{array}$$

where the *Wet_i,t_* and *Rice_i,t_*are the area of wetland and rice paddy, and the *N_w,i,t_* indicates the number of GWFG at the site *i* time *t*.

Bird movement was driven by migration pressure *P_ij_* between site *i* and all the other connected sites *j*, selecting the site with the highest pressure:

[3] $$\begin{array}{c} P_{ij,t}=\left\{\begin{array}{c} \frac{A_{j,t}-A_{i,t}}{R_{\mathrm{ij}}},ifA_{i,t}\geq 0,A_{j,t}\geq 0,and\;A_{j,t}-A_{i,t}>0\\ 0,otherwise \end{array}\right., \end{array}$$

where *R_ij_* is the resistance between sites, indicating the difficulty of flying over the distance:

[4] $$R_{\mathrm{ij}}=\frac{D_{\mathrm{ij}}}{D_{\mathrm{wb}}},$$

*D_ij_*is the distance between sites *i* and *j*, and *D_wb_*is the distance between the northernmost breeding and southernmost wintering sites. Birds, therefore, choose destinations based on resource availability and distance. Furthermore, the number of birds at a site was calculated as

[5] $$N_{w,i,t}=N_{w,i,t-1}+\theta \times \sum_{j=1}^{q}M_{w,ji,t-1}\times s_{m}-\sum_{j=1}^{q}M_{w,ij,t},$$

where *θ* is the fraction of migrants arriving in time step *t*, *s_m_* is the survival rate, $\sum_{j=1}^{q}M_{w,ji,t-1}$ describes birds arriving from other sites, and $\sum_{j=1}^{q}M_{w,ij,t}$ describes birds departing site *i* (see detailed description in *SI Appendix*, *Method* S1).

#### Simulating pathogen transmission.

We integrated SIR models to simulate the transmission of two LPAIV strains: LPAIV_w_ (originating in wild birds) and LPAIV_p_ (originating in poultry). We assumed that wild birds were infection-free at breeding sites (73) and first acquired LPAIV_w_ at stopover sites (18), initiating circulation within the wild population (74).

We focused on the viral diversitification and subtype emergence in poultry, assuming a positive correlation with reassortment following coinfection by two strains. This focus reflects evidence that poultry populations often serve as incubators for novel strains (1, 9). Additionally, spillover from wild waterfowl into poultry occurs more frequently than the reverse, and it is a main driver of virus spread (56). Thus, to simplify the model, we simulated the unidirectional spillover of LPAIV_w_ from wild birds to poultry, while acknowledging that bidirectional viral exchange can occur in reality (1, 9).

**Fig. 6.**
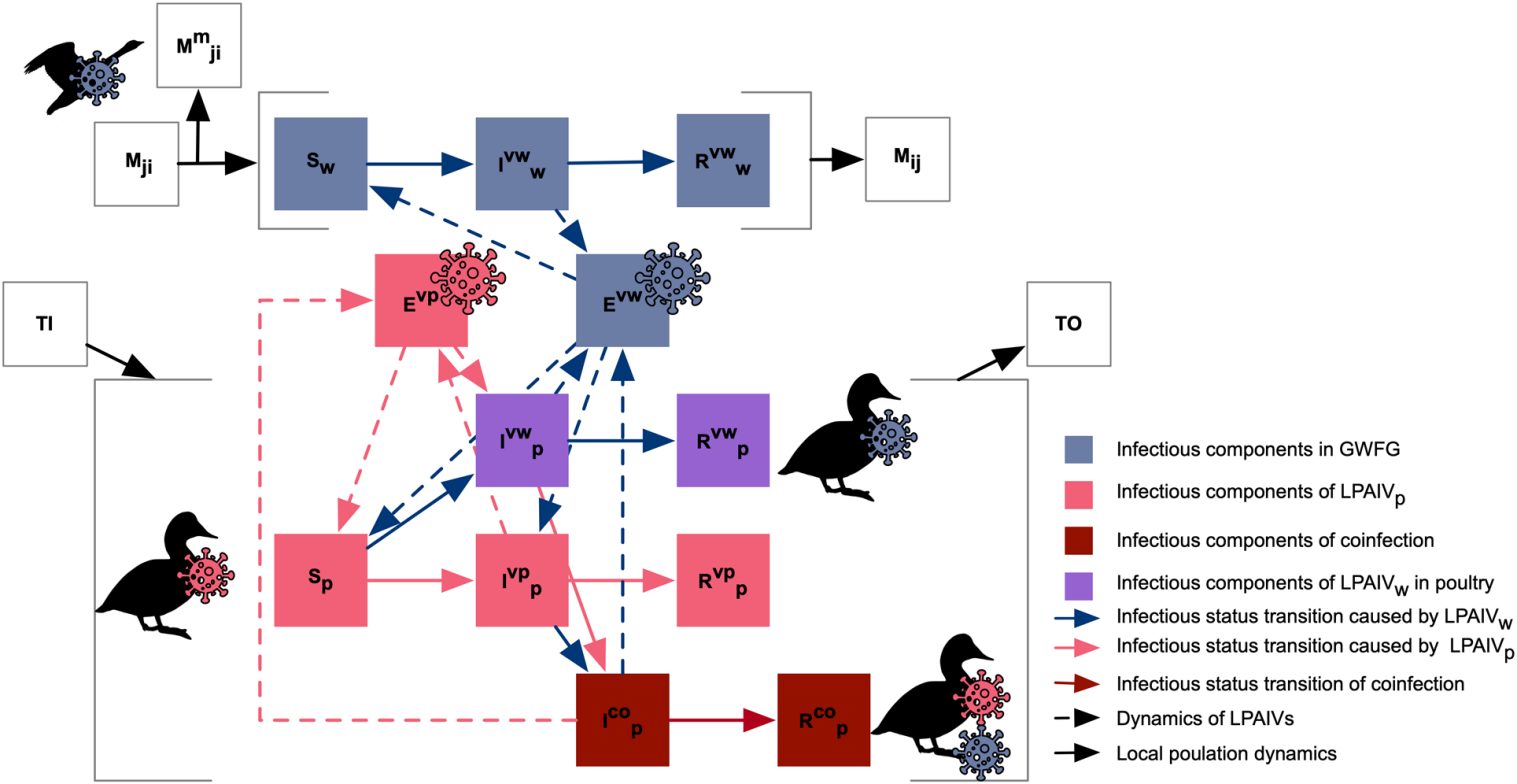
Conceptual model of LPAIV_w_ and LPAIV_p_ transmission in host populations. Blocks of S, I, R, and E indicate the components of susceptible, infected, and recovered individuals and viruses in the environment; blocks of M, TI, and TO indicate the components of migration, trade-in, and trade-out. The subscripts of ij, w, and *p* indicate the pair of sites, wild birds, and poultry birds. The superscripts of m, vw, vp, and co indicate the mortality, LPAIV_w_, LPAIV_p_, and coinfection.

In our model, when migrating wild birds reach a site containing poultry, all local poultry birds are considered susceptible to LPAIV_w_, regardless of any existing LPAIV_p_ infection. When exposed to LPAIV_w_, susceptible poultry (*S_p_*) and those recovered from LPAIV_p_ (*R_p_^vp^*) move to component *I_p_^vw^* (Fig. 6), while poultry already infected with LPAIV_p_ (*I_p_^vp^*) can move to the coinfection compartment (*I_p_^co^*), with susceptibility reduced by partial immunity (*ρ*). Coinfected poultry (*I_p_^co^*) enables reassortment between wild- and poultry-origin strains, and consequently, we calculated the reassortment incidence as a proxy to indicate the risk of viral diversification and novel subtype emergence. We modeled only LPAIV transmission, assuming all infections are asymptomatic. This approach avoids the complexities of simulating HPAIV-associated pathogenicity, mortality, detection, and control measures (e.g., culling), which were beyond the scope of this study. In addition, poultry population change through periodic trade-outs (TO), followed one day later by trade-ins (TI) of uninfected birds (Fig. 6; and see detailed transmission dynamics and their mathematical descriptions in *SI Appendix*, *Method S2*).

### Parameterization.

#### Bird migration parameters.

We estimated GWFG migration parameters by averaging seasonal data from each tagged individual, including migration duration (*M_duri_*), migration distance (*M_dist_*), resting duration on stopovers (*R_duri_*), flying duration in migration (*F_duri_*), flying speed (*F_speed_*), number of stopover sites (*N_stop_*), and step length (*S_length_*). Migration duration was calculated as the elapsed time between the first and last timestamp of a seasonal track, and migration distance was the distance between the southernmost breeding and northernmost winter sites. We summed the elapsed time among the GPS locations inside the 50% dBBMM contour to obtain the resting duration and summed the elapsed time outside the contour for flying duration. Flying speed was calculated as the cumulative distance divided by flying duration; the number of stopover sites was directly counted from the dBBMM results; and step length was estimated by dividing the migration distance by the number of stopover sites. Additional parameters, including population size of wild birds (*N_w_*), species body mass (*B_mass_*) and its SD (*B_massSD_*), body mass accumulation and consumption rates (*A_mass_* and *C_mass_*), body mass threshold for starting migration (*T_mass_*), and survival rate during migration (*s_m_*), were taken from previous studies that were carried out in the same region (27, 72, 75) (see values of migration parameters and their sources in *SI Appendix*, Table S7).

#### Poultry population dynamic parameters.

The size of the poultry population (*N_p_*) at each site was estimated as the product of resources and poultry density (see Datasets S2 and S3 for years 2000 and 2015, respectively). The trading interval (i.e., duration between two trading events, *T_inter_*), and trade-in/out volumes (*TI* and *TO*) were selected to represent a typical intensive broiler duck raising cycle and management practices in the study region (76) (see values of poultry dynamic parameters and their sources in *SI Appendix*, Table S8).

#### Disease transmission parameters.

To simplify the model, we used the same epidemiological parameters for the transmission of LPAIV_w_ and LPAIV_p_. Despite parameters of virus transmission coefficient (*β*), viral decay rate (*ε*), and infectious duration (*1/γ*), we introduced four parameters to regulate the spillover, coinfection, and reassortment processes in the model: cross-species transmission rate (*σ*), efficacy of partial immunity (*ρ*), contribution of coinfection to LPAIV_p_ infection (*ϕ*; i.e., probability that a coinfected bird transmits LPAIV_p_ onward), and reassortment efficiency (*τ*). Furthermore, to parameterize the initial infection condition for the poultry populations in each site, we preran the sole transmission of LPAIV_p_ in poultry for 1,000 steps in each scenario (*SI Appendix*, Fig. S11) and used the most frequent infection condition from dynamic equilibrium (*SI Appendix*, Fig. S12) (see detailed parameterization in the *SI Appendix*, *Method* S3; see values of transmission parameters and their sources in *SI Appendix*, Table S9).

#### Individual-based model scenarios.

The simulations began when infection-free GWFG initiated body mass gain at breeding sites, and initial LPAIV_w_ exposure occurred upon first arrival at stopovers, while poultry started with the background prevalence. Additionally, we replicated the simulations with varied first LPAIV_w_ exposure, either at breeding sites or at wintering sites, reflecting studies that suggest wild birds may carry the infection during breeding (17) and/or acquire it upon arrival at wintering grounds (18, 73) (*SI Appendix*, Fig. S13). We also tested the model’s sensitivity to the viral decay rate (*ε*) and cross-species transmission rate (*σ*) by varying their values ±10%, respectively, to create 9 × 9 combinations. The risk of emergence from each parameter combination was compared to that of the default scenario (*SI Appendix*, Fig. S7). In this study, each scenario was run for 3,000 iterations of 150 daily time steps, a sufficient time window for fall migration and overwintering, with outputs averaged to generate final results.

#### Simulation outputs and analysis.

We exported the migration trajectories and number of visiting birds at each site and time step to generate movement networks and evaluate the accuracy of the spatial distribution against dBBMM results (*SI Appendix*, Table S10). We also exported daily counts of surviving wild birds in each infectious class, as well as new infections from direct transmission. For poultry, we calculated population size by infectious class and environmental viral loads from both wild and poultry birds. To indicate the risk of diversification and novel subtype emergence, we calculated the cumulative reassortment incidences and the rates of reassortment scaled by the simulation steps.

With the migration networks generated with GLM sites, we calculated various network-level metrics to indicate overall connectivity (*SI Appendix*, Table S1), and site-level centrality metric betweenness to indicate the role of each site in bird migration (Datasets S2 and S3). Using the IBM simulation results, we constructed migration networks to illustrate the birds’ spatial distribution (i.e., number of GWFG that visited each site), and compared migration parameters (i.e., number of days resting, number of days flying, number of stops, and migration duration) between the years 2000 and 2015 (*SI Appendix*, Fig. S14).

We used piecewiseSEM to analyze how environmental variables influence bird distribution and cumulative reassortment at each site, using nested path analysis to quantify the unique effect of each predictor and prevent potential collinearity (77). We first constructed full models that included all the associations between the predictors and response variables that were mathematically programmed in our model (*SI Appendix*, Fig. S15). After that, we created 22,785 nested models for bird distribution and 87,885 nested models for reassortment for the 2000 and 2015 scenarios, respectively, by randomly removing one to all associations from the full models. Finally, we assessed the goodness of fit of each nested model by comparing the Akaike information criterion (AIC) among the models with Fisher’s C *P*-value smaller than 0.05, reporting the model with the lowest AIC value (77) (*SI Appendix*, Figs. S13 and S14 for the top nested models with ∆AIC ≤ 2 of bird distribution in 2000 and 2015, and *SI Appendix*, Figs. S15 and S16 of reassortment).

To visualize the spatial changes in the potential risks, we mapped the difference in reassortment incidence between 2000 and 2015. To account for varying exposure timings during migration, we averaged cumulative reassortment incidence across different exposure scenarios for each habitat site and assigned this value to the irregular water body polygon represented by that site. Next, we overlaid a 1° × 1° hexagonal grid and averaged site-level incidence within each cell for both years (see the risk maps for 2000 and 2015 in *SI Appendix*, Figs. S17 and S18). The final map presents the change in the risks as the difference in hexagon-level incidences between the years.

In this study, the remote sensing data were processed in an open-source geographic information system (QGIS version 3.22) (78), the IBM was built and simulated in an agent-based modeling platform (NetLogo version 6.1.1) (71), and the data were processed and analyzed with a statistical language (R version 4.4.1) (79). The eBird data were processed by using the package “*auk*” (67), and the dBBMM and piecewiseSEM analyses were implemented by using the packages “*move*” (80) and “*piecewiseSEM*” (81), respectively.

## Supplementary Material

Appendix 01 (PDF)

## Data Availability

All study data, code scripts, and model are included in the article, supporting information, and Figshare: https://doi.org/10.6084/m9.figshare.28352081.v1 (82).
