# Supplementary material for: Landscape changes elevate the risk of avian influenza virus diversification and emergence in the East Asian–Australasian Flyway
Source: Proc Natl Acad Sci U S A. 2025 Aug 18;122(34):e2503427122. doi: 10.1073/pnas.2503427122 (PMC12403075; doi:10.1073/pnas.2503427122)
Supplement: Supplementary file 1 — Appendix 01 (PDF) [file pnas.2503427122.sapp.pdf]

PNAS

Supporting Information for

Landscape changes elevate risk of avian influenza virus diversification and emergence in the East Asian-Australasian Flyway

Shenglai Yin, Chenchen Zhang, Claire S. Teitelbaum, Yali Si, Geli Zhang, Xinxin Wang, Dehua Mao, Zheng Y.X. Huang, Willem Frederik de Boer, John Takekawa, Diann J. Prosser, Xiangming Xiao

Shenglai Yin and Diann J. Prosser
Email: shenglai.yin@ou.edu and dprosser@usgs.gov

Any use of trade, firm, or product names is for descriptive purposes only and does not imply endorsement by the U.S. Government.

This PDF file includes:

Methods S1 to S3
Figs. S1 to S21
Tables S1 to S10
SI References

Other supporting materials for this manuscript, including datasets, individual-based model, and R scripts, are available at Figshare: 10.6084/m9.figshare.28352081

Dataset S1 to S4
Model S1
Code S1

Method S1

The behavior rule for simulating Greater White-fronted goose fall migration in the individual-based model.

This Method S1 outlines the decision-making process of the simulated Greater White-fronted geese (GWFG, *Anser albifrons*) in the individual-based model (IBM). In the diagram below, white boxes indicate TRUE/FALSE decision points, while green boxes represent action steps. Blue arrows denote TRUE outcomes, red arrows indicate FALSE outcomes, and black arrows represent sequential actions. Octagonal symbols mark events: T represents the termination of the simulation, R denotes the removal of an individual, and E indicates the end of actions for a given time step (1 time step equivalent to 1 day). The goose silhouette was sourced from https://www.phylopic.org/.

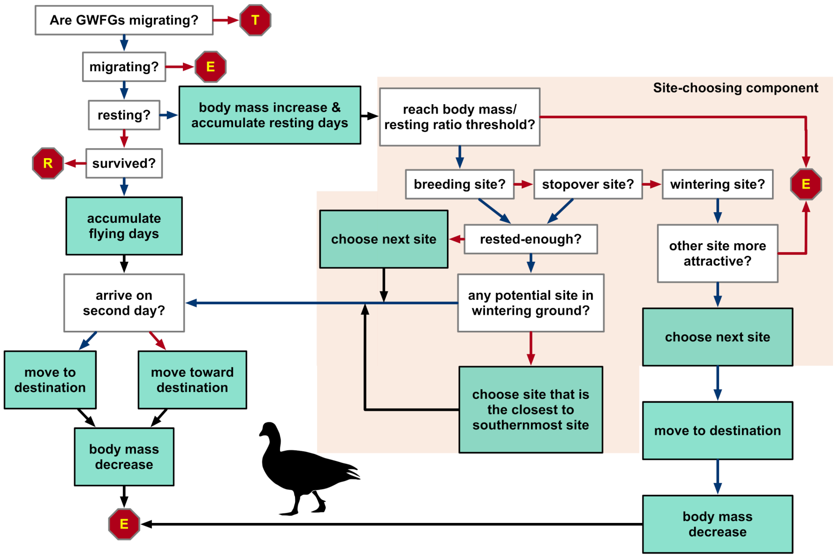

***SI Appendix* Fig. S1 Behavior rules of GWFG (agents) in the Individual-Based Model.**

At the start of the simulation, 10,000 migratory GWFG were generated and proportionally distributed across breeding sites. Each individual was assigned an initial body mass ($B_{mass}(0)$) drawn from a normal distribution $N(B_{mass}, B_{mass}SD^2)$, where B_{mass} represents the average body mass of GWFG and $B_{mass}SD$ is the standard deviation. Each goose was also assigned a binomial variable, **"*migrating?*"**, to indicate its migration status (TRUE/FALSE). Migration was initiated (*migrating? = TRUE*) when a GWFG's body mass reached the migration threshold (T_{mass}) at any breeding site. The migration status switched off (*migrating? = FALSE*) upon arrival at the final wintering site or when no further movement was possible. Additionally, each individual was assigned another binomial variable, **"*resting?*"**, to indicate whether it was flying (*resting? = FALSE*) or resting at a site (*resting? = TRUE*). The resting status was set to FALSE during flight between sites and TRUE while on the ground. Each GWFG was also assigned several numeric variables, including the expected number of stopover sites (N_{stop}), flying speed (F_{fly}), body mass (B_{mass}), body mass accumulation rate (A_{mass}), and body mass consumption rate (C_{mass}). The decision-making process for each individual is described in detail below.

At each simulation time step, the IBM model first performs a global inspection to determine whether the simulation should terminate. This occurs if all GWFGs have completed their migration (*i.e., migrating? = FALSE* for all individuals). Once this inspection is completed, each GWFG follows its designated decision-making rules to execute behaviors for that time step.

If the GWFG is resting (*resting? = TRUE*), it gains body mass and accumulates its resting duration while proceeding to the site-selection decision-making process, "*site-choosing component*". If the GWFG is not resting (*resting? = FALSE*), the model first assesses whether the individual survives migration based on the survival rate (S_{mig}). Surviving individuals then update their flight duration (F_{duri}) and, depending on the distance to the next stopover site, either move directly to the destination or move one day's movement distance (km/day) toward it. Before the time step ends, the GWFG's body mass

decreases due to energy expenditure during flight.

Site-choosing component:

A resting GWFG that does not meet the condition for flying (i.e., its body mass remains below the migration threshold T_{mass}) will end its simulation at that time step. Otherwise, its behavior depends on its current location. If the GWFG is at a breeding or stopover site and has not yet stopped at enough sites ($< N_{stop} - 1$), it selects the next stopping location based on migration pressure, choosing the site with the highest pressure link to its current location (see equation (1) in the manuscript). However, if the GWFG has already stopped at enough sites ($\geq N_{stop} - 1$), it either selects a connected wintering site (if available) or moves to the closest site to the southernmost wintering site if no direct wintering sites are connected.

A GWFG that arrives at its first wintering site evaluates whether any other wintering site is more attractive by comparing attractiveness values (A_j). If no more attractive site is found, the GWFG remains at its current location for the rest of the simulation. Otherwise, it selects a final wintering site based on migration pressure, moves there directly, and decreases its body mass accordingly.
The parameter values and their sources are summarized in *SI Appendix*, Table S7.

Method S2

Mathematical descriptions of the epidemiological process in the individual-based model.

This Method S2 presents the mathematical equations for the epidemiological dynamics illustrated in the conceptual model (Fig. 6 in the main manuscript). The complete model consists of four key components: (1) $LPAIV_w$ transmission within the wild bird population, (2) $LPAIV_p$ transmission within poultry populations, (3) $LPAIV_w$ spillover from wild to poultry populations, and (4) coinfection with both $LPAIV_w$ and $LPAIV_p$ in poultry, as well as the reassortment incidences calculated from coinfection.

LPAIV transmission in both wild and poultry populations follows an SIR model with an indirect environmental transmission pathway. The core transmission dynamics among the susceptible (S), infected (I), and recovered (R) compartments, along with the environmental viral load (E), are described by the following equations:

1. $S_{i,t} = S_{i,t-1} - \beta \times S_{i,t-1} \times \left(I_{i,t-1} + E_{i,t-1}\right)$
2. $I_{i,t} = I_{i,t-1} + \beta \times S_{i,t-1} \times \left(I_{i,t-1} + E_{i,t-1}\right) - \gamma \times I_{i,t-1}$
3. $R_{i,t} = R_{i,t-1} + \gamma \times I_{i,t-1}$

The subscripts *i* and *t* represent the location and simulation time step, respectively (these subscripts apply to the following equations). The parameter *β* denotes the transmission coefficient, while *γ* represents the recovery rate. The environmental viral load (E) is treated as an equivalent number of infectious birds by converting it into a bird-scale metric (see equations 4–6) (1).

Since the viral load (*V*) in the environment is influenced by viral shedding from infected birds (*η*) and natural decay (*ε*), its dynamics can be described as:

4. $V_{i,t} = V_{i,t-1} - \varepsilon \times V_{i,t-1} + \eta \times I_{i,t-1} - \varepsilon \times \eta \times I_{i,t-1}$

To express the viral load in terms of an equivalent number of infectious birds, we divide equation 4 by the shedding rate *η*, yielding:

5. $\frac{V_{i,t}}{\eta} = \frac{V_{i,t-1}}{\eta} - \varepsilon \times \frac{V_{i,t-1}}{\eta} + I_{i,t-1} - \varepsilon \times I_{i,t-1}$

We then introduced a new parameter, $E_{i,t}$, to replace $\frac{V_{i,t}}{\eta}$ (i.e., the unit is $\frac{viron}{viron/_{bird}} = bird$, simplified to an equivalent number of infectious birds). This transformation allows us to express the dynamics of environmental viral load in terms of bird equivalents, removing the explicit dependence on the virus shedding rate *η*, thus

6. $E_{i,t} = E_{i,t-1} - \varepsilon \times E_{i,t-1} + I_{i,t-1} - \varepsilon \times I_{i,t-1}$

Equations 1-3 and 6 serve as the foundational models for describing the dynamics of each compartment in our model. These equations are integrated with population changes driven by migration in wild birds and trading events in poultry populations.

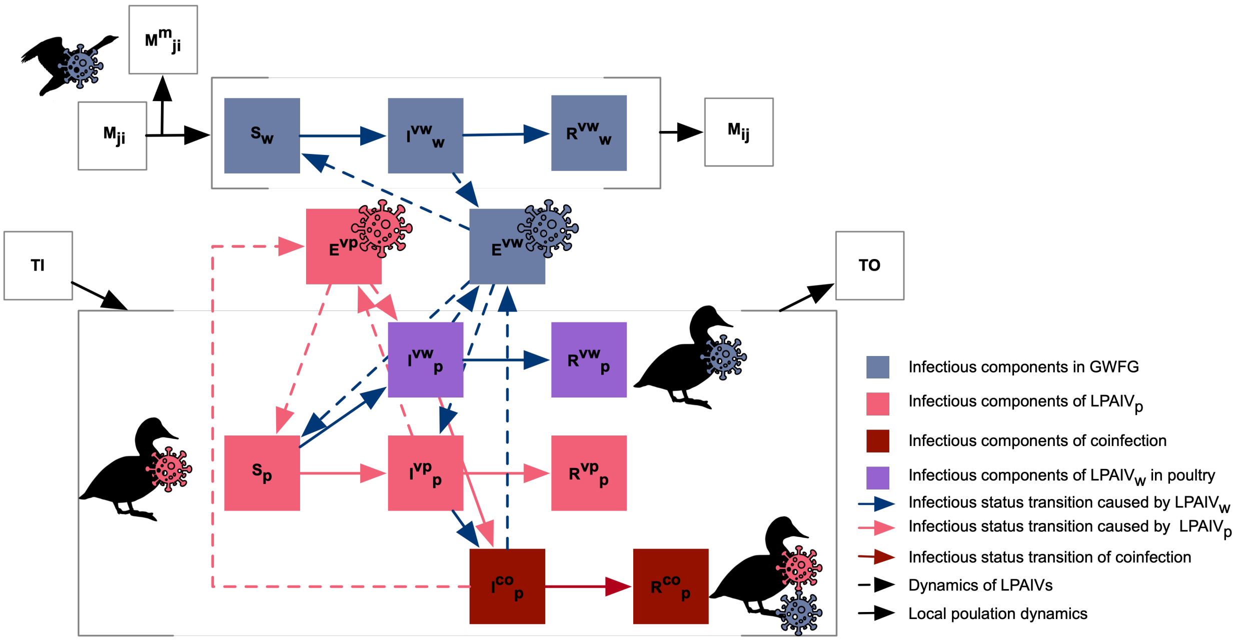

***SI Appendix*, Fig. S2 Conceptual model of $LPAIV_w$ and $LPAIV_p$ transmission in host populations (Fig. 6 in the main manuscript).** Blocks of S, I, R, and E indicate the components of susceptible, infected, and recovered individuals and viruses in the environment; blocks of M, TI, and TO indicate the components of migration, trade-in, and trade-out. The subscripts of *ij*, *w*, and *p* indicate the pair of sites, wild birds, and poultry birds. The superscripts of *m*, *vw*, *vp*, and *co* indicate the mortality, $LPAIV_w$, $LPAIV_p$, and coinfection, respectively.

The population dynamics of susceptible wild birds at site *i* (S_W) are influenced by migration, including individuals arriving from other sites *j*, departing from site *i*, and transitioning from susceptible to infected status. Therefore, S_W, is expressed as:

7. $$S_{w,i,t} = S_{w,i,t-1} + \theta \times \sum_{j=1}^{q} MS_{w,ji,t-1} \times s_m - S_{w,i,t}^{S\to I} - \sum_{j=1}^{q} MS_{w,ij,t-1}$$

where $\sum_{j=1}^{q} MS_{w,ji,t-1}$ represents the total number of susceptible wild birds departing from various sites *j* and arriving at site *i* at time step *t-1*. However, the actual number reaching site *i* depends on two factors: the migration survival rate (s_m) and the proportion of birds (θ) that can complete the journey within a single time step. The remaining proportion (*1-θ*) represents individuals whose departure sites are beyond a one-day travel distance, meaning they will arrive at site *i* in later time steps. Additionally, $\sum_{j=1}^{q} MS_{w,ij,t-1}$ represents the total number of susceptible birds departing from site *i* to other sites *j*, while $S_{w,i,t}^{S\to I}$ denotes the number of wild birds transitioning from susceptible to infected status.

8. $$S_{w,i,t}^{S\to I} = \beta \times S_{w,i,t-1} \times \left(I_{w,i,t-1}^{vw} + E_{i,t-1}^{vw}\right)$$

$I^{vw}_{w,i,t-1}$ represents the number of infected wild birds, while $E^{vw}_{i,t-1}$ denotes the environmental viral load of $LPAIV_w$ shed by wild birds.

In our model, the total environmental viral load of $LPAIV_w$, E^{vw}, consists of two components: the viral load shed by wild birds, E^{vw}_{w}, and the viral load shed by poultry birds infected with $LPAIV_w$, E^{vw}_{p}. Both E^{vw}_{w} and E^{vw}_{p} contribute to the transmission of $LPAIV_w$, but E^{vw}_{p} is additionally affected by clearance due to poultry trading events (see equations 21–22).

Therefore, the total environmental viral load of $LPAIV_w$, ($E^{vw}_{i,t}$), at site *i* and time step *t* is given by:

9. $$E_{i,t}^{vw} = E_{w,i,t}^{vw} + E_{p,i,t}^{vw}$$

Similarly to the equation 6, the $E^{vw}_{w,i,t}$ is calculated as

10. $$E_{w,i,t}^{vw} = E_{w,i,t-1}^{vw} - \varepsilon \times E_{w,i,t-1}^{vw} + I_{w,i,t-1}^{vw} - \varepsilon \times I_{w,i,t-1}^{vw}$$

The number of infected wild birds, ($I^{vw}{}_{w}$), is influenced by multiple factors. It increases with the number of infected wild birds arriving at site *i* from other sites *j*, ($\theta \times \sum_{j=1}^{q} MI_{w,ji,t-1} \times s_m$), and decreases due to newly recovered birds $I_{w,i,t}^{vw,I\to R}$, newly infected birds transitioning from susceptible to infected $S_{w,i,t}^{S\to I}$, and the infected wild birds departing from site *i* to other sites *j*, $\sum_{j=1}^{q} MI_{w,ij,t-1}^{vw}$. Thus, $I^{vw}{}_{w,i,t}$ is calculated as:

11. $I_{w,i,t}^{vw} = I_{w,i,t-1}^{vw} + \theta \times \sum_{j=1}^{q} MI_{w,ji,t-1} \times s_m + S_{w,i,t-1}^{S\to I} - I_{w,i,t-1}^{vw,I\to R} - \sum_{j=1}^{q} MI_{w,ij,t-1}^{vw}$, and
12. $I_{w,i,t}^{vw,I\to R} = \gamma \times I_{w,i,t-1}^{vw}$

Similarly, the dynamic of recovered wild birds, $R^{vw}{}_{w,i,t}$, is

13. $R_{w,i,t}^{vw} = R_{w,i,t-1}^{vw} + \theta \times \sum_{j=1}^{q} MR_{w,ji,t-1} \times s_m + I_{w,i,t-1}^{vw,I\to R} - \sum_{j=1}^{q} MR_{w,ij,t-1}^{vw}$

The above mathematical descriptions are bout the $LPAIV_w$ in the wild bird population, whereas the below descriptions are about $LPAIV_w$ and $LPAIV_p$ in poultry populations. LPAIV transmission in poultry involves three key processes: the spread of $LPAIV_p$ within poultry populations, the spillover of $LPAIV_w$ from wild to poultry birds, as well as the dynamics of coinfection and reassortment. The poultry population at site *i* is periodically regulated by trade-out, (*TO*) and trade-in (*TI*) events. All trade-in birds are assumed to be susceptible, while trade-out birds are proportionally removed from each poultry compartment (*SI Appendix*, Fig. S2). To simulate periodic trading, we assume trade-out events occur every T_{inter} time step (day) during the simulation:

14. $TO_{i,t} = \begin{cases} -T, when\ t = \alpha \times T_{inter}, \alpha \in \mathbb{Z}_{\geq 1} \\ 0, else \end{cases}$

Trade-in occurs on the second day following each trade-out event, with the trade-in volume fully compensating for the population loss from trade-out:

15. $TI_{i,t} = \begin{cases} T, when\ t = \alpha \times T_{inter} + 1, \alpha \in \mathbb{Z}_{\geq 1} \\ 0, else \end{cases}$

Apart from trading events, the number of susceptible poultry birds decreases as individuals become infected with either $LPAIV_w$, $S^{S_p \to I_p^{vw}}$, or $LPAIV_p$, $S^{S_p \to I_p^{vp}}$. Therefore, the dynamics of susceptible poultry birds can be described as:

16. $S_{p,i,t} = S_{p,i,t-1} + TI_{i,t-1} - S^{S_p \to I_p^{vw}} - S^{S_p \to I_p^{vp}} - \vartheta_{i,t-1} \times S_{p,i,t-1}$

where ϑ represents the proportion of trade-out within the total poultry population.

17. $\vartheta_{i,t} = \frac{T}{S_{p,i,t-1} + I_{p,i,t-1}^{vp} + R_{p,i,t-1}^{vp} + I_{p,i,t-1}^{vw} + R_{p,i,t-1}^{vw} + I_{p,i,t-1}^{co} + R_{p,i,t-1}^{co}}$
18. $S^{S_p \to I_p^{vw}} = \beta \times S_{p,i,t-1} \times \sigma \times \left(I_{w,i,t-1}^{vw} + I_{p,i,t-1}^{vw} + E_{i,t-1}^{vw} + (1-\phi) \times I_{p,i,t-1}^{co}\right)$

Equation 18 describes the conversion of poultry from the susceptible compartment to the infected with $LPAIV_w$ compartment. This process is driven by transmission from wild birds infected with $LPAIV_w$ ($I^{vw}{}_{w}$), poultry infected with $LPAIV_w$ ($I^{vw}{}_{p}$), the environmental viral load of $LPAIV_w$ (E^{vw}), and coinfected poultry ($I^{co}{}_{p}$). To account for cross-species transmission, we introduce the parameter σ, representing the cross-species transmission rate. Additionally, we incorporate a theoretical parameter, ϕ, to indicate the contribution of coinfected poultry to $LPAIV_p$ transmission, where 1-ϕ represents the proportion of coinfected poultry contributing to $LPAIV_w$ transmission.

Similarly, the number of susceptible poultry converted to infected with $LPAIV_p$, $S^{S_p \to I_p^{vp}}$, in equation 16 is calculated as:

19. $S^{S_p \to I_p^{vp}} = \beta \times S_{p,i,t-1} \times (I_{p,i,t-1}^{vp} + E_{i,t-1}^{vp} + \phi \times I_{p,i,t-1}^{co})$

We did not include the cross-species transmission rate, σ, in equation 19 because it describes $LPAIV_p$ transmission solely among poultry hosts and does not involve cross-species transmission. In this equation, E^{vp} represents the environmental viral load of $LPAIV_p$, which is influenced by virus shedding from infected poultry and clearance due to trading events. Thus, E^{vp} can be calculated as:

20. $E_{i,t}^{vp} = \begin{cases} E_{i,t-1}^{vp} \times (1 - \vartheta_{i,t-1}), when\ t = \alpha \times k, \alpha \in \mathbb{Z}_{\geq 1} \\ E_{i,t-1}^{vp} + (I_{p,i,t-1}^{vp} + \phi \times I_{p,i,t-1}^{co}) - \varepsilon \times E_{i,t-1}^{vp} - \varepsilon \times I_{p,i,t-1}^{vp} + \phi \times I_{p,i,t-1}^{co}), else \end{cases}$

In addition, the environmental viral load of $LPAIV_w$ shed by poultry birds, (E^{wp}), is also subjected to the clearance associated with trading events. Thus, the E^{wp} is calculated as

21. $E_{(i,t)}^{wp} =$
$\begin{cases} E_{i,t-1}^{wp} \times (1 - \vartheta_{i,t-1}), when\ t = \alpha \times k, \alpha \in \mathbb{Z}_{\geq 1} \\ E_{i,t-1}^{wp} + \left(I_{p,i,t-1}^{vw} + (1 - \vartheta_{i,t-1}) \times I_{p,i,t-i}^{co}\right) - \varepsilon \times E_{i,t-1}^{wp} - \varepsilon \times \left(I_{p,i,t-1}^{vw} + (1 - \vartheta_{i,t-1}) \times I_{p,i,t-i}^{co}\right), else \end{cases}$

The dynamic of poultry birds infected $LPAIV_p$ (I_p^{vp}), is calculated as

22. $I_{p,i,t}^{vp} = I_{pi,t-1}^{vp} + S_{p,i,t-1}^{S_p \to I_p^{vp}} - I_{p,i,t-1}^{vp \to co} - \gamma \times I_{p,i,t-1}^{vp} - \vartheta_{i,t-1} \times I_{p,i,t-1}^{vp}$

The $I_{p,i,t}^{vp \to co}$ represents the number of poultry birds initially infected with $LPAIV_p$ that acquire $LPAIV_w$ and become coinfected, entering the I_p^{co} component:

23. $I_{p,(i,t)}^{vp \to co} = \beta \times \rho \times I_{p,i,t-1}^{vp} \times \left(I_{w,i,t-1}^{vw} + I_{p,i,t-1}^{vw} + E_{i,t-1}^{vw} + (1 - \phi) \times I_{p,i,t-1}^{co}\right)$

The parameter ρ is the partial immunity rate associated with a previous infection (either $LPAIV_w$ or $LPAIV_p$).

The dynamic of poultry birds recovered from $LPAIV_p$ infection is calculated as

24. $R_{p,i,t}^{vp} = R_{p,i,t-1}^{vp} + \gamma \times I_{p,i,t-1}^{vp} - \vartheta_{i,t-1} \times R_{p,i,t-1}^{vp}$

The dynamic of poultry birds infected with $LPAIV_w$, ($I^{vw}{}_p$), is positively influenced by new infected birds, and negatively influenced by the recovered birds, trading events, and the conversion into coinfection. Thus, I_p^{vw} is calculated as

25. $I_{p,i,t}^{vw} = I_{p,i,t-1}^{vw} + S_{p,i,t-1}^{S_p \to I_p^{vw}} + R_{p,i,t-1}^{R_p \to I_p^{vw}} - I_{p,i,t-1}^{I_p^{vw} \to I_p^{co}} - I_{p,i,t-1}^{I_p^{vw} \to R_p^{vw}} - \vartheta_{i,t-1} \times I_{p,i,t-1}^{vw}$
26. $S_{p,i,t}^{S_p \to I_p^{vw}} = \beta \times \rho \times S_{p,i,t-1} \times \left(I_{p,i,t-1}^{vw} + I_{w,i,t-1}^{vw} + E_{i,t-1}^{vw} + (1 - \phi) \times I_{p,i,t-1}^{co}\right)$
27. $R_{p,(i,t)}^{R_p \to I_p^{vw}} = \beta \times \rho \times \sigma \times R_{p,i,t-1}^{vp} \times \left(I_{p,i,t-1}^{vw} + I_{w,i,t-1}^{vw} + E_{i,t-1}^{vw} + (1 - \phi) \times I_{p,i,t-1}^{co}\right)$

Since the poultry birds infected with $LPAIV_w$ ($I^{vw}{}_p$), can further infect $LPAIV_p$ and enter the component of $I^{co}{}_p$, or recover to enter the component $R^{vw}{}_p$, thus

28. $I_{p,i,t}^{I_p^{vw} \to I_p^{co}} = \beta \times \rho \times I_{p,i,t-1}^{vw} \times (I_{p,i,t-1}^{vp} + E_{i,t-1}^{vp} + \phi \times I_{p,i,t-1}^{co})$
29. $I_{p,i,t}^{I_p^{vw} \to R_p^{vw}} = \gamma \times I_{p,i,t-1}^{vw}$

The compartment of poultry birds with coinfection is positively influenced by the conversion of $I^{vw}{}_p$ and $I^{vp}{}_p$, and negatively influenced by the recovery and the trading events. Thus,

30. $I_{p,i,t}^{co} = I_{p,i,t-1}^{co} + I_{p,i,t-1}^{I_p^{vw} \to I_p^{co}} + I_{p,i,t-1}^{I_p^{vp} \to I_p^{co}} - I_{p,i,t-1}^{I_p^{co} \to R_p^{co}} - \vartheta_{i,t-1} \times I_{p,i,t-1}^{co}$
31. $I_{p,i,t}^{I_p^{vp} \to I_p^{co}} = \beta \times \sigma \times I_{p,i,t-1}^{vp} \times \left(I_{w,i,t-1}^{vw} + I_{p,i,t-1}^{vw} + E_{i,t-1}^{vw} + (1 - \phi) \times I_{p,i,t-1}^{co}\right)$
32. $I_{p,i,t}^{I_p^{co} \to R_p^{co}} = \gamma \times I_{p,i,t-1}^{co}$

And the recovery components in the poultry population are

33. $R_{p,i,t}^{vw} = R_{pi,t-1}^{vw} + \gamma \times I_{p,i,t-1}^{vw} - \vartheta_{i,t-1} \times R_{p,i,t-1}^{vw}$
34. $R_{p,i,t}^{co} = R_{p,i,t-1}^{co} + \gamma \times I_{p,i,t-1}^{co} - \vartheta_{i,t-1} \times R_{p,i,t-1}^{co}$

Eventually, we calculate the reassortment incidence as

34. $Re_{i,t} = I_{p,i,t-1}^{co} \times \tau$

Where τ is the reassortment efficiency.

Method S3

Parameterization of disease transmission processes in the individual-based model.
To simplify the model, we used the same epidemiological parameters for the transmission of $LPAIV_w$ and $LPAIV_p$. The virus transmission coefficient (β) was estimated as the minimal value that can cause $LPAIV_w$ transmission in a non-migrating wild bird population (i.e., the β can lead to $R_0 = 1$ in the population) with 1% initial infected individuals (2), and the viral decay rate (ε), infectious duration ($1/\gamma$) were extracted from previous studies (*SI Appendix*, Table S9).

We introduced four parameters to regulate the spillover, coinfection, and reassortment processes in the model: cross-species transmission rate (σ), efficacy of partial immunity (ρ), contribution of coinfection to $LPAIV_p$ infection (ϕ), and reassortment efficiency (τ). The cross-species transmission rate (σ) played a role when $LPAIV_w$ infected poultry, and the parameter was estimated as the proportion of cross-species transmission events from wild hosts to poultry hosts that occurred in Asia between 1974 and 2014. The cross-species transmission events were identified following a previous study that used the reappearance of two AIV sequences with a >99% similarity sampled from two different species groups in the same region in less than 3 years (3). The efficacy of partial immunity (ρ) applies when $LPAIV_w$ infects poultry that were already infected with a strain or recovered from an infection, and the parameter was extracted from previous studies (4, 5). The contribution of coinfection to $LPAIV_p$ infection (ϕ) was set as a theoretical value representing the probability that a coinfected poultry individual transmits $LPAIV_p$ to other poultry.

Eventually, we estimated the reassortment efficiency (τ) using statistically supported reassortment rates for the six internal gene segments (PB2, PB1, PA, NP, M, NS). These rates were derived from paired donor-recipient subtypes which were estimated in a previous study (6). We calculated minimum, mean, and maximum estimates for the overall efficiency by combining the corresponding rates across these six segments. To incorporate uncertainty in this parameter, we used a truncated normal distribution to represent its probable values. We set the mean of this distribution to our calculated overall mean. The distribution's range was defined by our calculated overall minimum and maximum estimates. We used a theoretical standard deviation of 0.02 to define the width of the normal distribution (*SI Appendix*, Fig. S3).

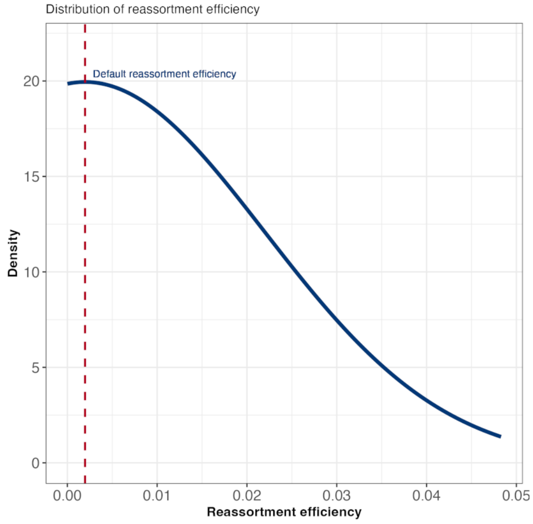

***SI Appendix*, Fig. S3 Probability density of the reassortment efficiency τ.**

For each IBM simulation run, we randomly drew 10,000 values from this distribution and used the mean as the reassortment efficiency τ for that run. This approach aimed to integrate the parameter's uncertainty into the simulation and reflect it in the outcomes. The parameters for LPAIVs transmission in GWFG and poultry populations are listed in *SI Appendix*, Table S9.

Furthermore, we considered the poultry populations as groups in which the infection of $LPAIV_p$ can form a dynamic equilibrium, and we aim to use the most common infection prevalence as the initial infection status for the simulations. Thus, we pre-ran the sole transmission of $LPAIV_p$ in poultry for 1000 steps in each scenario (*SI Appendix*, Fig. S11). With the simulated outputs, we visually inspected for the

equilibrium and excluded results within the first six infection peaks and results after the last infection peak. We calculated the frequencies of the prevalence combinations and used the most frequent combination as the initial infection condition for the poultry populations in each site (*SI Appendix*, Fig. S11).

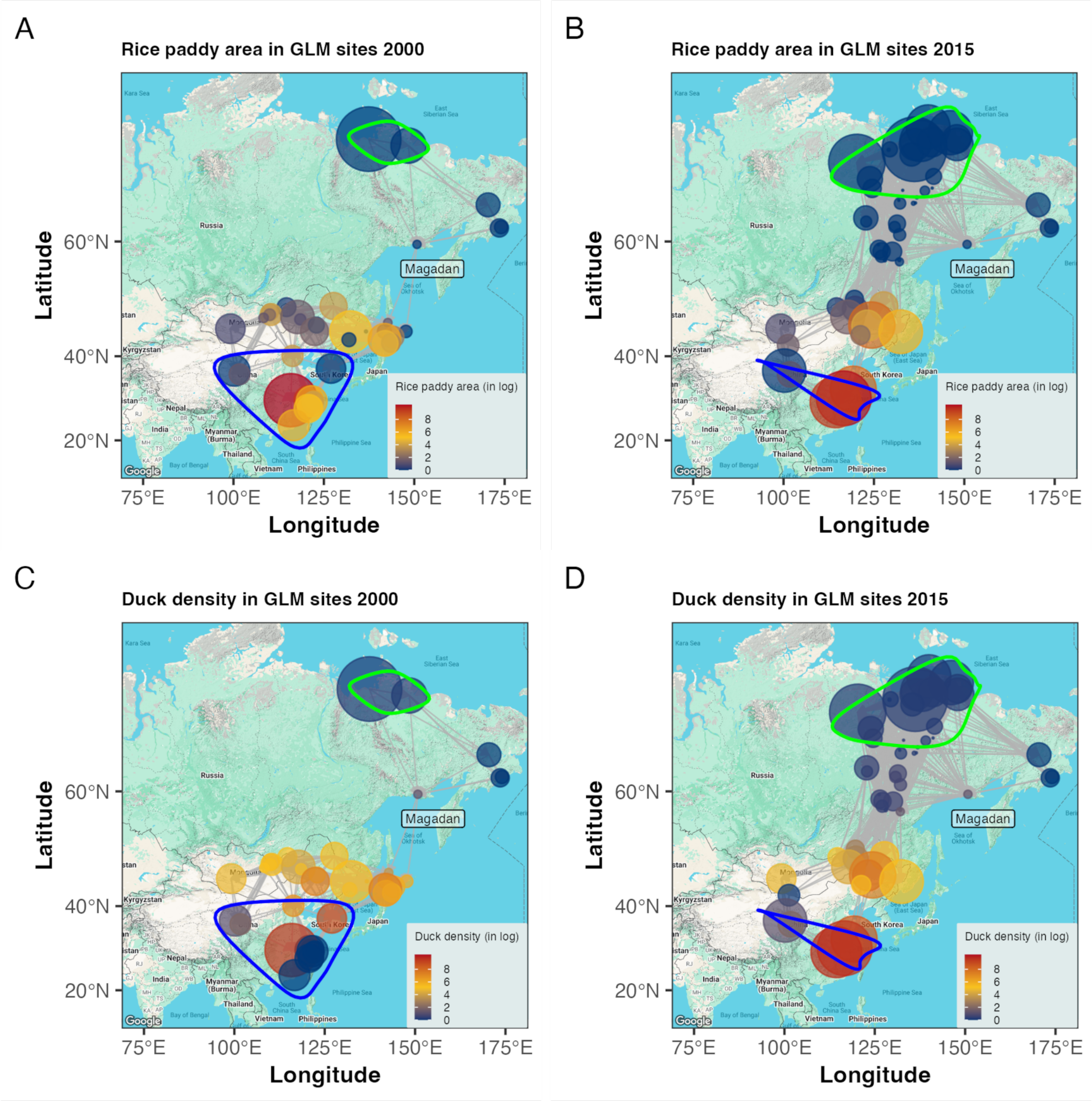

Fig. S4 Rice paddy area and poultry density across GLM predicted sites in years 2000 and 2015. **(**A) rice paddy area across sites in 2000; (B) rice paddy area across sites in 2015**;** (C) poultry density across sites in 2000; and (D) poultry density across sites in 2015. The rice paddy area (km^2) and poultry density (individuals per km^2) were log-transformed. The base maps were extracted from the R package "*ggmap*".

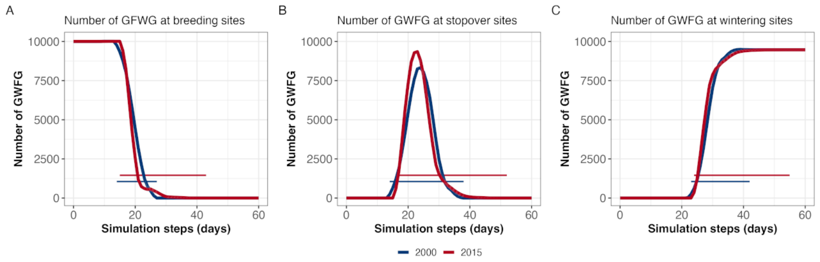

Fig. S5 Number of Greater white-fronted goose (GWFG) at breeding, stopover, and wintering sites over the simulation period. (A) number of GWFG at breeding sites; (B) number of GWFG at stopover sites; and (C) number of GWFG at wintering sites. The curves indicate the number of GWFG, ribbons indicate the standard deviations, and the horizontal segments indicate the period when GWFG is present at the sites. The outputs were averaged over 3000 simulations from the individual-based model.

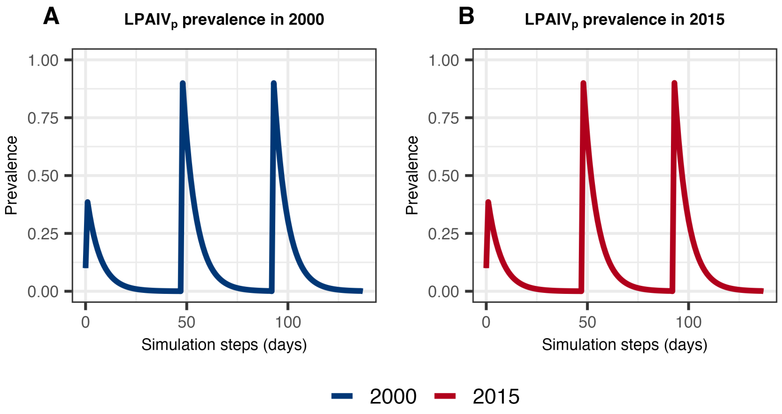

Fig. S6 Dynamics of LPAIV$_p$ infection prevalence in the poultry population. (A) Infection prevalence in 2000, and (B) infection prevalence in 2015. The outputs were averaged over 3000 simulations from the individual-based model.

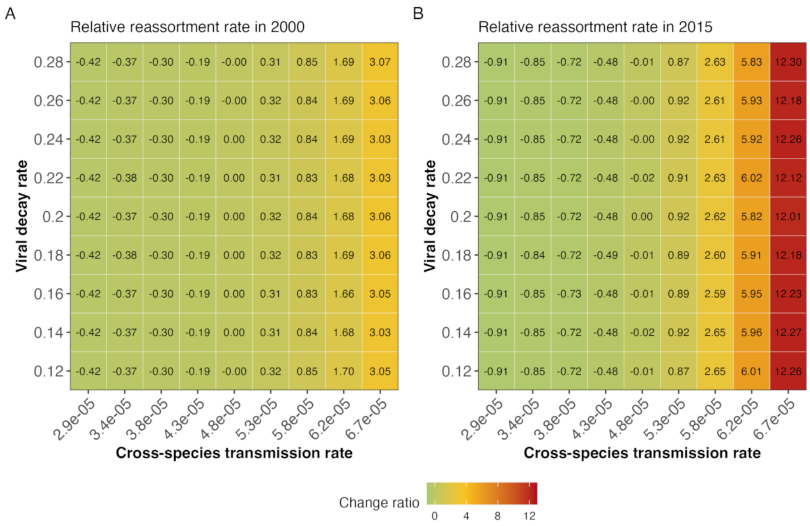

Fig. S7 Relative reassortment rate (reassortment incidence per day) in various scenarios of viral decay rate and cross-species transmission rate combinations. (A) relative reassortment rate in 2000, and (B) relative reassortment rate in 2015. The reassortment rate was calculated for each scenario, and the change ratio was calculated by comparing its reassortment rate to the default scenario at the center of each panel (viral decay rate = 0.2 and cross-species transmission rate = $4.8e^{-5}$). The outputs were averaged over 3000 simulations from the individual-based model.

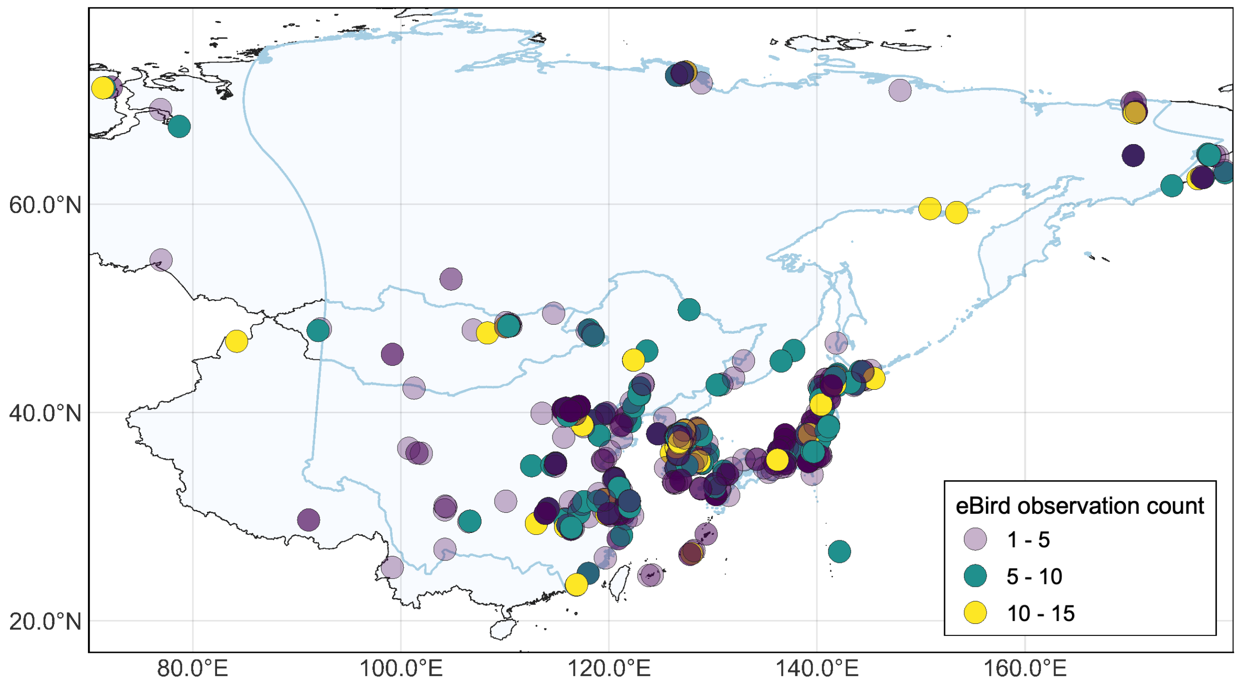

Fig. S8 Illustration of Greater White-fronted goose (GWFG) observation from eBird data in our study region from 1995 to 2020. The dot colors indicate the observation count, and the light blue polygon represents the study region in the East Asian-Australisian Flyway. The background of national and regional boundaries was sourced from the Generic Mapping Tools (GMT) Version 6.

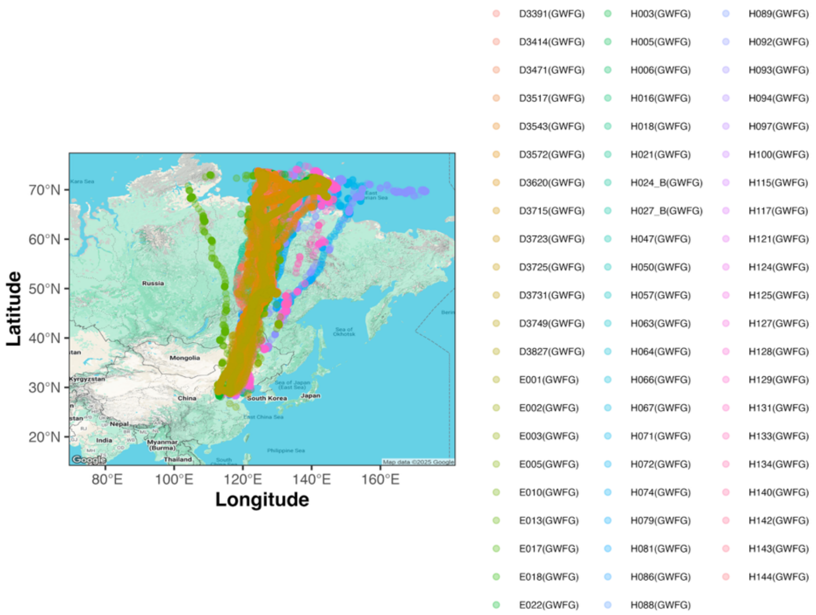

Fig. S9 Illustration of the telemetry tracking data of Greater White-fronted goose (GWFG) from 2014 to 2016. The dot colors indicate the tracked individuals. In total, 79 GWFG individuals were tagged at Poyang Lake (29.1˚N, 116.3˚E) in the winter of 2014/15. Tracking data collected from late 2014 to 2016 were used for this study, but only data from individuals who returned tracking locations were included. The base maps were extracted from the R package “*ggmap*”.

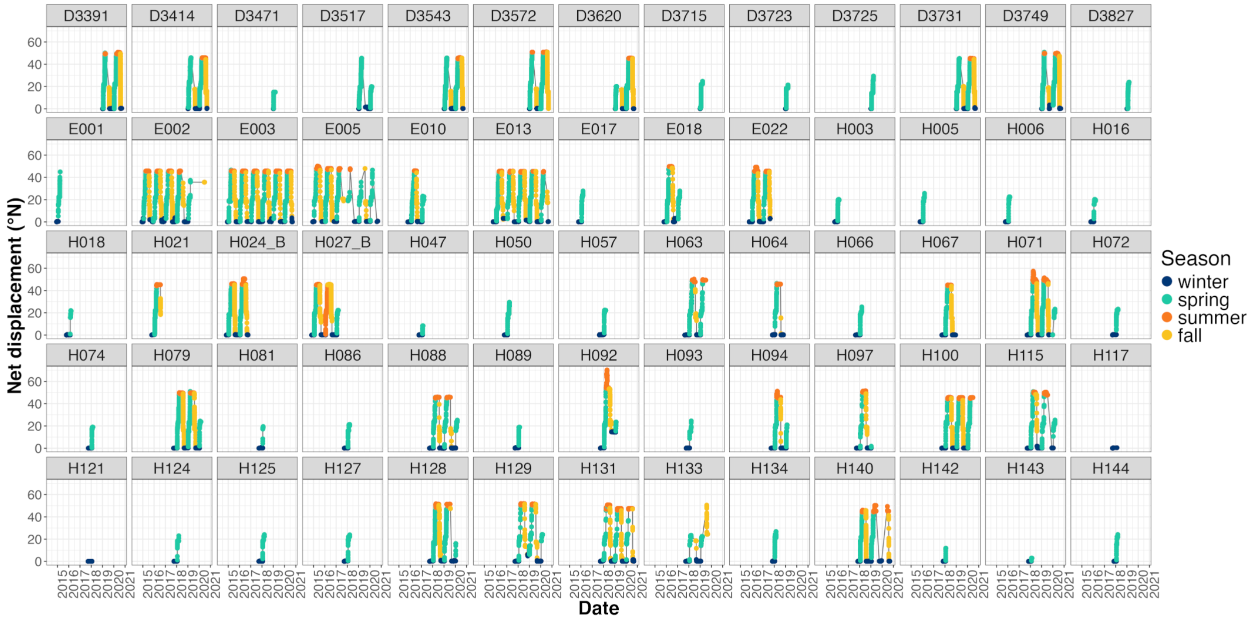

Fig. S10 Net displacement plot for all tracked Greater White-fronted geese (GWFG). This figure shows all returned GPS tracking data, including segments excluded from later analysis. Data from individuals that did not return any locations are omitted. The panel titles are the identifiers for GWFG individuals, and the segment colors represent seasons. The yellow segments extending from the orange summer segments to the blue winter segment represent the complete segments of fall migration, which were included in the analysis.

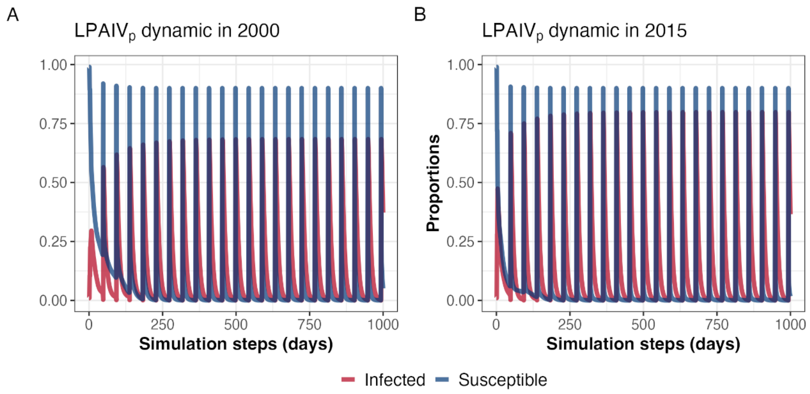

Fig. S11 Transmission dynamics from the pre-runs of $LPAIV_p$ in poultry. The results were simulated without spillover of $LPAIV_w$ or coinfection. The trade-in/out events occurred every 45 days for 90% of the population on each site.

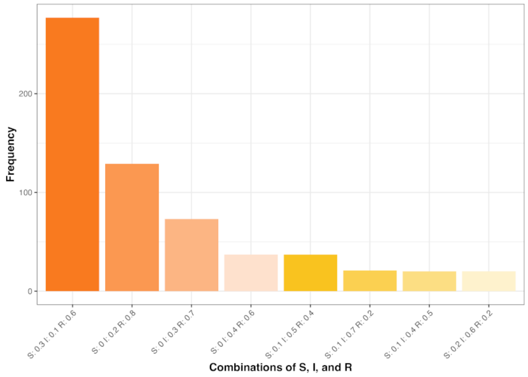

Fig. S12 Frequencies of the most common combinations among the infectious classes of susceptible, infected, and recovered in poultry. The values were extracted from the data of *SI Appendix*, Fig. S11, rounding to 1 digit. The top 5 most common combinations were extracted for 2015 and 2000, respectively, and we pooled the combinations since the scenarios shared the most common combinations. The combinations are shown as susceptible: infected: recovered on the x-axis.

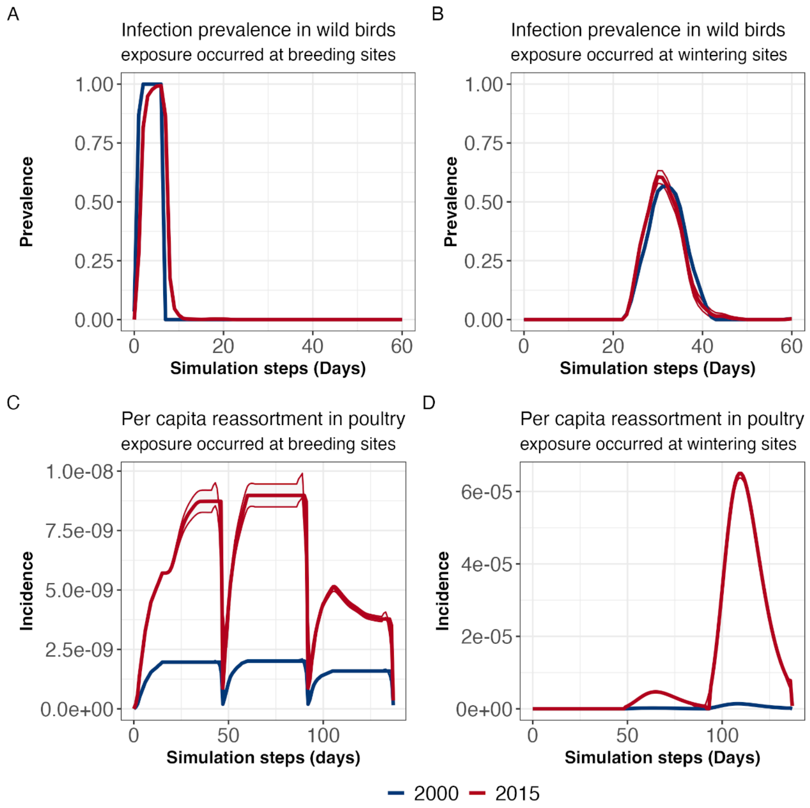

Fig. S13 Dynamics of LPAIV$_w$ prevalence in wild birds and reassortment in poultry from the scenarios of exposure timing. (A) dynamics of LPAIV$_w$ prevalence in wild birds when the first LPAIV$_w$ exposure occurred at breeding sites; (B) dynamics of LPAIV$_w$ prevalence when the exposure occurred at wintering sites; (C) dynamics of reassortment in poultry when the exposure occurred at breeding sites; (D) dynamics of reassortment in poultry when the exposure occurred at wintering sites. The outputs were averaged over 3000 simulations from the individual-based model.

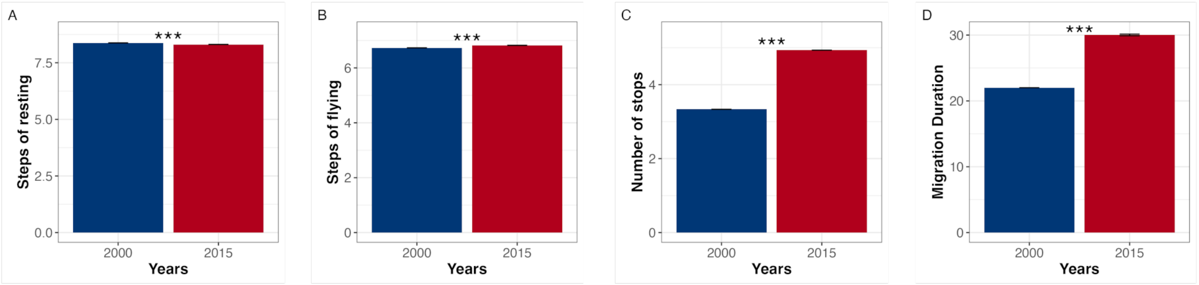

Fig. S14 Migration parameters generated from individual-based model simulations for the years 2000 and 2015. *** indicate the significant differences at the level of p-value ≤ 0.001. The outputs were averaged over 3000 simulations from the individual-based model.

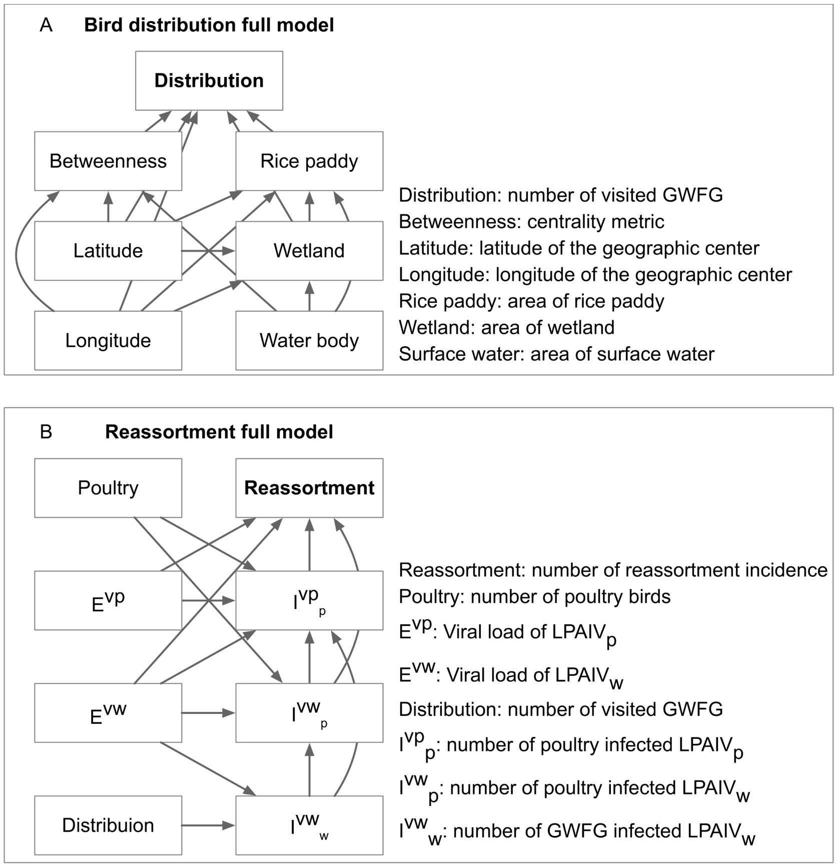

Fig. S15 Full models from the piecewise structural equation model analysis among sites in the individual-based model. (A) full model for bird distribution, and (B) full model for reassortment. The connections are built based on the mathematical descriptions embedded in the model (see *SI Appendix*, Method S2).

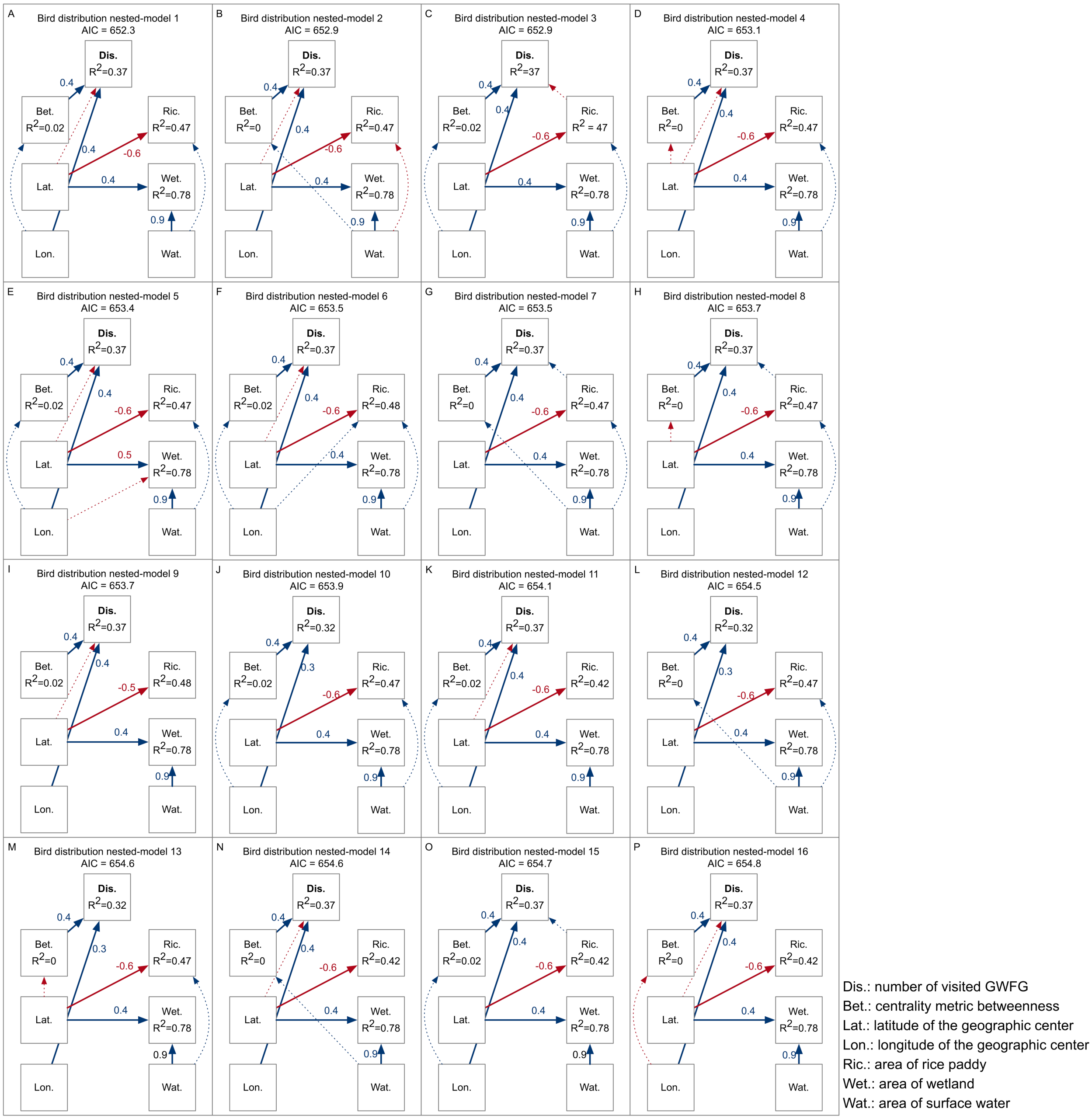

Dis.: number of visited GWFG
Bet.: centrality metric betweenness
Lat.: latitude of the geographic center
Lon.: longitude of the geographic center
Ric.: area of rice paddy
Wet.: area of wetland
Wat.: area of surface water

Fig. S16 Top nested models from the piecewise structural equation model analysis on wild bird distribution among sites in 2000. The arrows point from predictors to response variables. The solid arrows indicate significant impacts, the dashed arrows indicate insignificant impacts, whereas blue and red colors indicate positive and negative impacts. The effect sizes are annotated next to the arrows. The nested models were selected based on their Fisher's C p-value ≥ 0.05 and ΔAIC ≤ 2 to the optimal model in Fig. 2A.

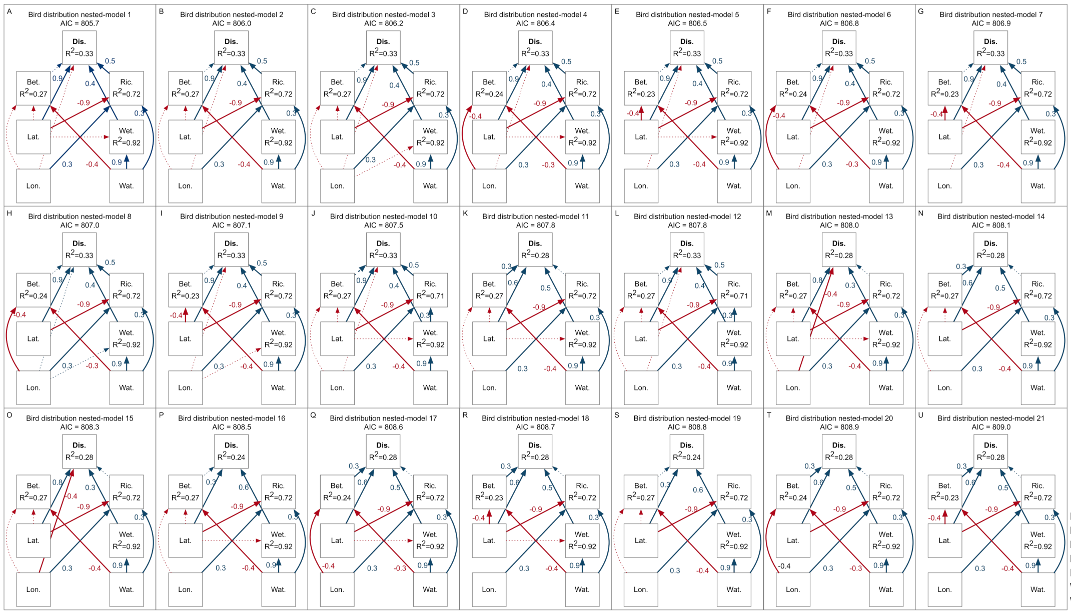

Dis.: number of visited GWFG
Bet.: centrality metric betweenness
Lat.: latitude of the geographic center
Lon.: longitude of the geographic center
Ric.: area of rice paddy
Wet.: area of wetland
Wat.: area of surface water

Fig. S17 Top nested models from the piecewise structural equation model analysis on wild bird distribution among sites in 2015. The arrows point from predictors to response variables. The solid arrows indicate significant impacts, the dashed arrows indicate insignificant impacts, whereas blue and red colors indicate positive and negative impacts. The effect sizes are annotated next to the arrows. The nested models were selected based on their Fisher's C p-value ≥ 0.05 and ΔAIC ≤ 2 to the optimal model in Fig. 2B.

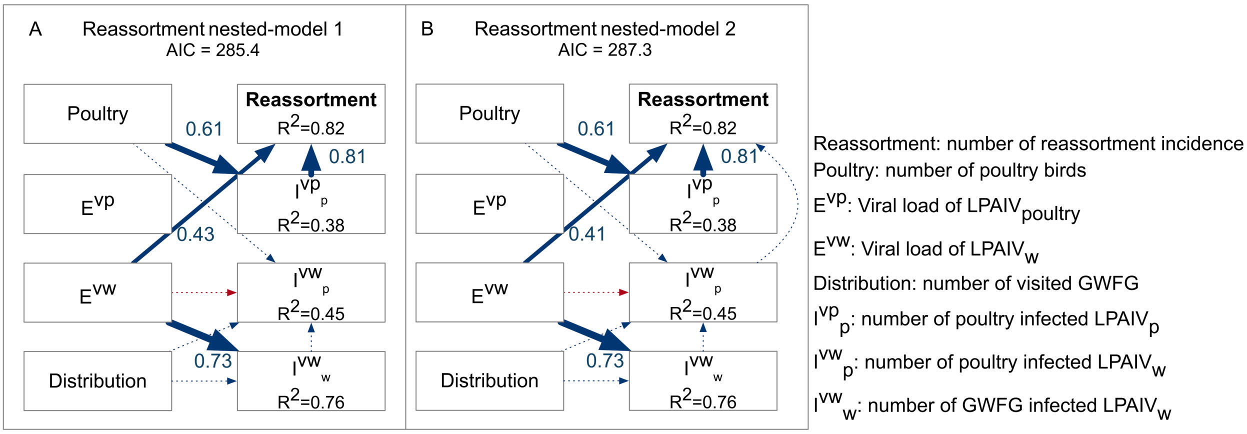

Reassortment: number of reassortment incidence
Poultry: number of poultry birds
E^{vp}: Viral load of $LPAIV_{poultry}$
E^{vw}: Viral load of $LPAIV_w$
Distribution: number of visited GWFG
$I^{vp}{}_p$: number of poultry infected $LPAIV_p$
$I^{vw}{}_p$: number of poultry infected $LPAIV_w$
$I^{vw}{}_w$: number of GWFG infected $LPAIV_w$

Fig. S18 Top nested models from the piecewise structural equation model analysis on reassortment among sites in 2000. The arrows point from predictors to response variables. The solid arrows indicate significant impacts, the dashed arrows indicate insignificant impacts, whereas blue and red colors indicate positive and negative impacts. The effect sizes are annotated next to the arrows. The nested models were selected based on their Fisher's C p-value ≥ 0.05 and ΔAIC ≤ 2 to the optimal model in Fig. 2C.

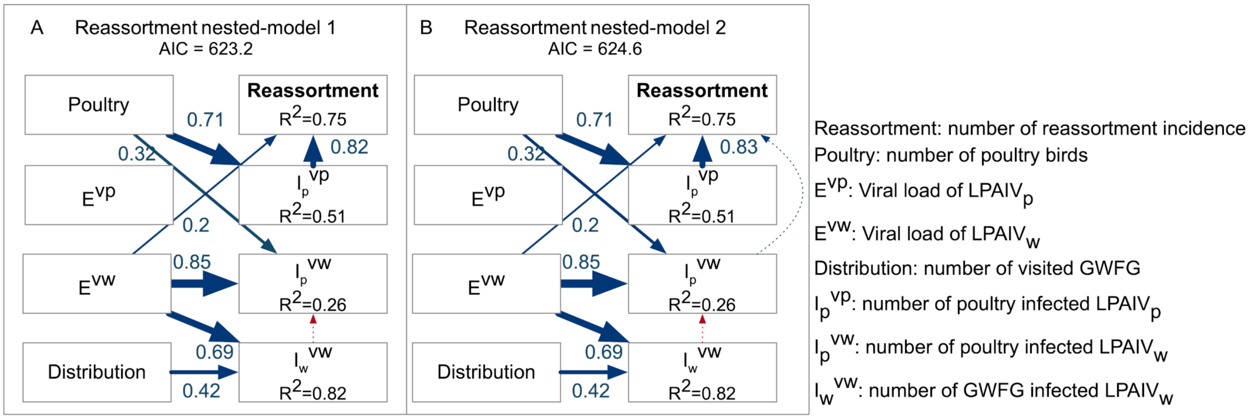

Fig. S19 Top nested models from the piecewise structural equation model analysis on reassortment among habitats in 2015. The arrows point from predictors to response variables. The solid arrows indicate significant impacts, the dashed arrows indicate insignificant impacts, whereas blue and red colors indicate positive and negative impacts. The effect sizes are annotated next to the arrows. The nested models were selected based on their Fisher's C p-value ≥ 0.05 and ΔAIC ≤ 2 to the optimal model in Fig. 2D.

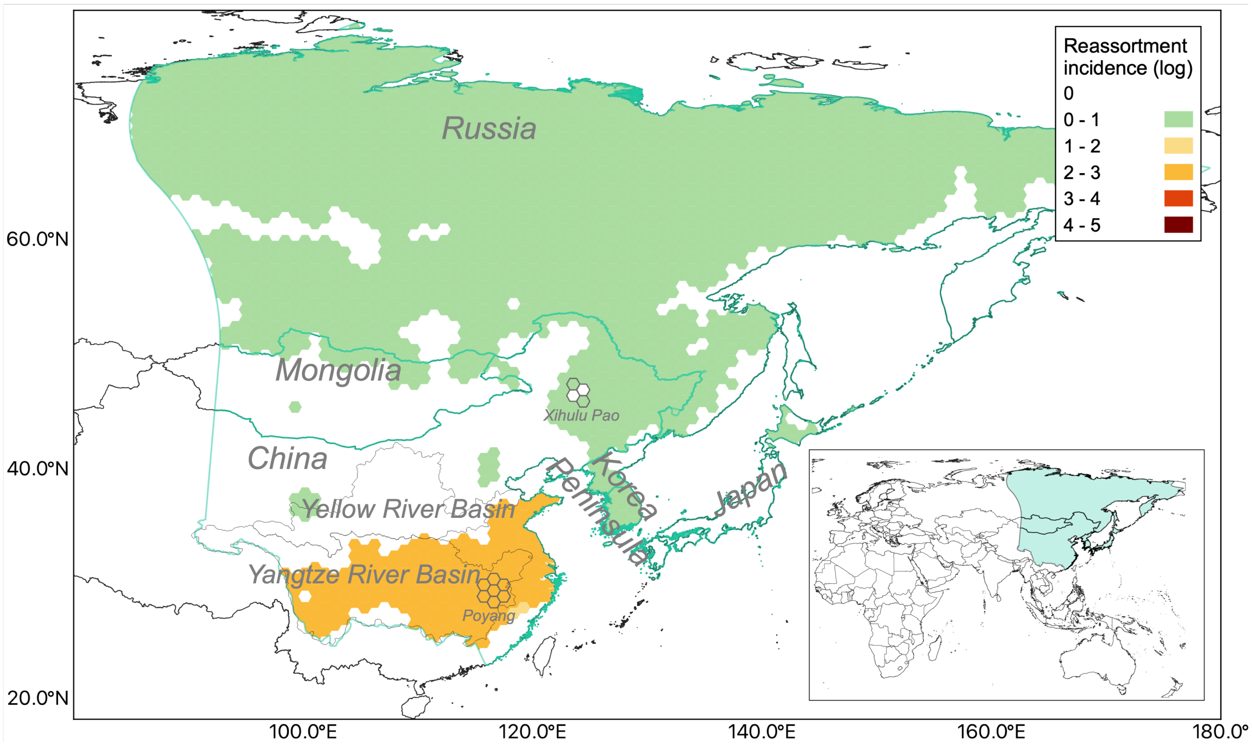

Fig. S20 Simulated reassortment incidences (in log) in 2000. The inset map provides geographic context showing the study region. Reassortment incidence is calculated per 1°×1° hexagon cell, and the outputs were averaged over 3000 simulations from the individual-based model. Light blue shaded area represents the study region boundary. Background maps sourced from Generic Mapping Tools (GMT) Version 6.

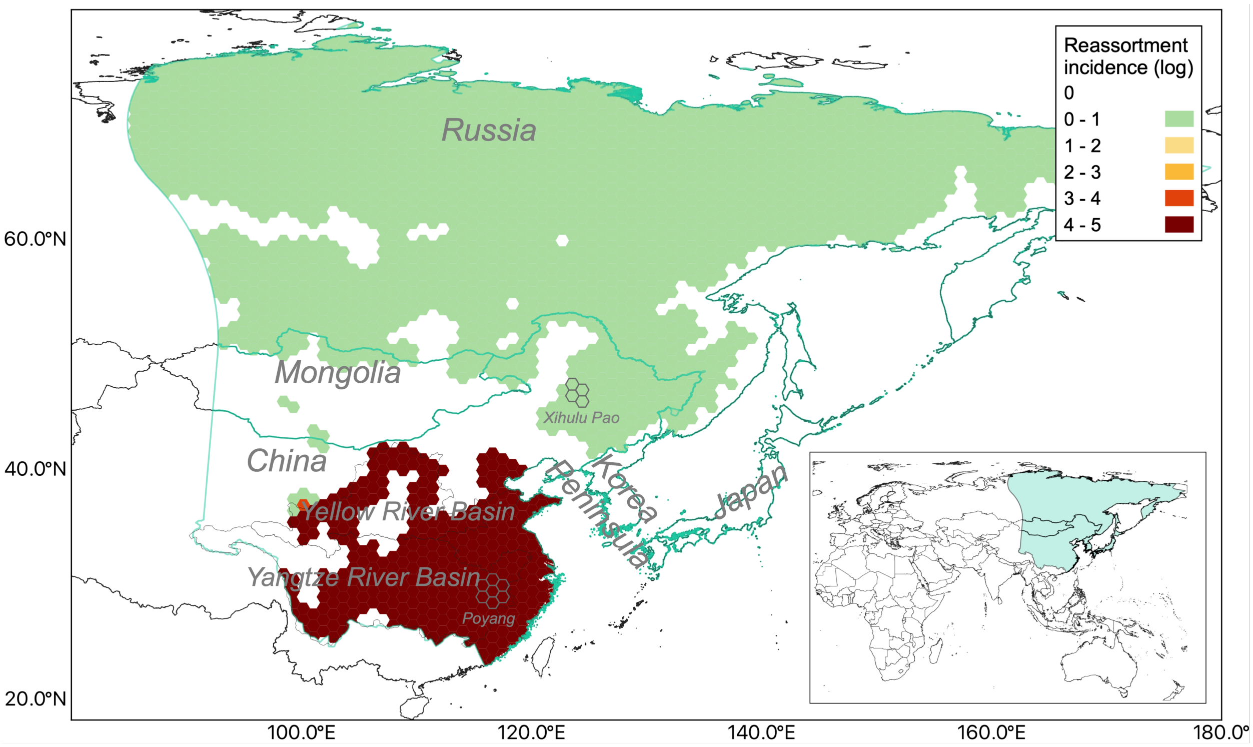

Fig. S21 Simulated reassortment incidences (in log) in 2015. The inset map provides geographic context showing the study region. Reassortment incidence is calculated per 1°×1° hexagon cell, and the outputs were averaged over 3000 simulations from the individual-based model. Light blue shaded area represents the study region boundary. Background maps sourced from Generic Mapping Tools (GMT) Version 6.

Table S1 Connectivity metrics of the networks of GLM predicted sites in 2000 and 2015. The metrics were calculated from the networks in Figs. 1B and D. Number of sites indicates the number of nodes in the network; density indicates the ratio of the number of actual links to the number of potential links in the network; average path length indicates the average length of the shortest paths between all pairs of sites; diameter indicates the longest shortest path between any two sites; clustering coefficient indicates a measurement of the sites' trend to cluster; efficiency indicates the average of the inverse of the shortest path lengths.

Year	Number of sites	Density	Average path length	Diameter	Clustering coefficient	Efficiency
2015	56	0.26	1.74	5	0.86	0.72
2000	41	0.15	2.15	6	0.74	0.58

Table S2 Results of the multiple GLM regression after bootstrapping. WatPeri is the water body perimeter, RiceArea is the rice paddy area, DuckDen is the domestic duck density, and HumDen is the human density. The multiple GLM was built with variables from the year 2015. Bold text indicates the *p-value* ≤ 0.05.

	b-value	95%CI lower	95%CI upper	p-value	z-value
Intercept	-0.49	-1.06	0.07	0.08	2.03
**Latitude**	**0.78**	**-0.02**	**1.58**	**0.04**	**-1.73**
**WatPeri**	**0.62**	**-0.08**	**1.33**	**0.01**	**1.59**
DuckDen	-0.73	-1.99	0.53	0.26	-1.13
HumDen	-0.26	-1.51	0.98	0.69	-0.40
RiceArea	0.11	-0.69	0.90	0.78	0.28

Table S3 Sources for the landscape and environmental data, telemetry tracking data, and eBird citizen science data.

#	Data	Data source	Year range
1	Surface water data inside China	Publication: Gainers and losers of surface and terrestrial water resources in China during 1989–2016 (7)	2000 and 2015
2	Surface water data outside China	Copernicus Climate Change Service. 2019 (8)	2000 and 2015
3	Wetland data inside China	Publication: National wetland mapping in China: A new product resulting from object-based and hierarchical classification of Landsat 8 OLI Images (9)	2000 and 2015
4	Wetland data outside China	Copernicus Climate Change Service. 2019 (8)	2000 and 2015
5	Rice paddy data inside China	Publication: Spatiotemporal patterns of paddy rice croplands in China and India from 2000 to 2015 (10)	2000 and 2015
6	Rice paddy data outside China	Copernicus Climate Change Service. 2019 (8)	2000 and 2015
7	Human density data	Center for International Earth Science Information Network – CIESIN. 2018 (11)	2000 and 2015
8	Road density data	Publication: Global patterns of current and future road infrastructure (12)	2018 (publishing year)
9	Domestic duck density data	Publication: Global distribution data for cattle, buffaloes, horses, sheep, goats, pigs, chickens and ducks in 2010 (13)	2010
10	eBird observation data	https://science.ebird.org/en/use-ebird-data/download-ebird-data-products (14)	1995-2022
11	Telemetry tracking data	Movebank study ID: 1614083125 (15)	2014-2016

Table S4 Results of the univariate logistic regression after bootstrapping. All values were averaged from 1000 repetitions of the bootstrapping procedure of the univariate regression. Bold predictors are significantly associated with the dependent variable and selected to construct the multiple GLM regression analysis (*SI Appendix*, Table S2). WatPeri is the surface water perimeter, WatArea is the surface water area, WetPeri is the wetland perimeter, WetArea is the wetland area, RiceArea is the rice paddy area, DuckDen is the domestic duck density, HumDen is the human density. The univariate logistic regression model is built with variables from the year 2015. Bold text indicates the *p-value* ≤ 0.05.

Predictor	Mean	SD	b-value	p-value	R^2
**WatPeri**	**386.54**	**2276.53**	**0.41**	**0.00**	**0.04**
WatArea	4995.74	42538.32	-0.11	0.50	0.00
WetPeri	2248.81	15665.99	0.22	0.14	0.01
WetArea	2123.38	23571.81	0.17	0.22	0.00
**RiceArea**	**253.93**	**1935.83**	**-0.97**	**0.00**	**0.10**
**DuckDen**	**7340.15**	**14910.03**	**-1.22**	**0.00**	**0.23**
**HumDen**	**546.24**	**1166.51**	**-1.24**	**0.00**	**0.20**
**Latitude**	**4048063.70**	**748216.48**	**1.63**	**0.00**	**0.32**
Longitude	14318368.78	1342754.68	-0.01	0.94	0.00

Table S5 Results of the independent variables correlation matrix. The bolded predictors are selected to construct the multiple GLM regression analysis (*SI Appendix*, Table S2). WatPeri is the surface water perimeter, WatArea is the surface water area, WetPeri is the wetland perimeter, WetArea is the wetland area, RiceArea is the rice paddy area, DuckDen is the domestic duck density, and HumDen is the human density. The correlation matrix is built with variables from the year 2015. Bold text indicates the variables selected for the final multiple GLM.

	WatArea	**WatPeri**	Longitude	**Latitude**	WetArea	WetPeri	**RiceArea**	**HumDen**	**DuckDen**
WatArea	1								
**WatPeri**	1	1							
Longitude	-0.15	-0.13	1						
**Latitude**	0.05	0.08	0.8	1					
WetArea	1	1	-0.13	0.08	1				
WetPeri	0.98	0.97	-0.17	0	0.97	1			
**RiceArea**	0.18	0.13	-0.28	-0.39	0.12	0.33	1		
**HumDen**	0.09	0.05	-0.5	-0.7	0.05	0.19	0.67	1	
**DuckDen**	0.07	0.04	-0.58	-0.79	0.03	0.17	0.69	0.68	1

Table S6 Accuracy of the multiple GLM regression in predicting the suitable habitat sites for Greater White-fronted goose (GWFG). GLM presence and GLM absence indicate sites classified by the multiple GLM analysis with the cutoff threshold of 0.6, and the dBBMM presence and dBBMM absence indicate the sites identified by the dynamic Brownian Bridge Movement Model with telemetry tracking data.

	GLM presence	GLM absence	Sum
dBBMM presence	39	11	50
dBBMM absence	15	161	176
Sum	54	172	226

Table S7 Parameters estimated from the telemetry tracking data and literature to simulate the Greater White-fronted goose (GWFG) migration in the individual-based model.

Parameter	Explaination	Value	Unit	Source
N_w	Wild bird population size	10,000	*individual*	(1, 16)
M_{dist}	Fall migration distance	5166 (4776 - 5436)	*km*	(17) and this study
M_{duri}	Duration (time elapse) of fall migration	35 (29 - 41)	*day*	(17) and this study
R_{duri}	Duration of resting in fall migration	21 (13 - 27)	*day*	(17) and this study
F_{duri}	Duration of flying in fall migration	14 (7 - 21)	*day*	(17) and this study
W_{duri}	Duration of overwintering phrase	103 (95 - 112)	*day*	this study
R_{rest}	Proportion of resting duration to the whole duration of fall migration	0.6 (0.6 - 0.7)	-	this study
F_{speed}	Flying speed in fall migration	577 (455 - 716)	*km/day*	(17) and this study
N_{stop}	Number of stopover sites in fall migration	3 (2 - 6)	-	(17) and this study
S_{length}	Step length in fall migration	1591 (1045 - 2222)	*km*	(17, 18) and this study
B_{mass}	Averaged body mass of GWF goose species	2538 (2075-3000)	*g*	(19)
B_{massSD}	Standard deviation of the averaged body mass of GWF goose species	215	*g*	(19)
B_{min}	Minmal body mass	2075	*g*	(19)
B_{max}	Maxmium body mass	3000	*g*	(19)
T_{mass}	Threshold of body mass for migration	15% (12%-17.5%)	-	(20)
A_{mass}	Body mass accumulation rate	24.6 (24.6-30)	*g/day*	(1, 20)
C_{mass}	Body mass consumption rate	0.1	*g/km*	(1)
s_m	Survival rate in fall migration	0.992 (0.9914-0.9971)	*/day*	(20, 21)
D_{wb}	Distance between the southmost breeding and northmost wintering sites	5006	*km*	this study
Res_{max}	Maximum value for the resources among the sites, and the resources were calculated as the sum of wetland area and rice paddy area (log-transformed)	12.8	*km^2*	this study
α	Scaling parameter to control the	-0.015	-	-

	shape of the function curve of attractiveness

Table S8 Parameters to simulate poultry population dynamics in the individual-based model.

Parameter	Explanation	Value	Unit	Source
N_p	Poultry population size	density×resources area	*bird*	this study
TO	Proportion of the trade-out to the population size	0.9	-	(22)
TI	To compensate the population reduction from the trade-out	0.9	-	(22)
T_{inter}	Duration between trade-in/out events (equivent to poultry duck farming cycle)	45	*day*	(22)
$S_{t=0}$	Initial proportion of susceptible birds	0.3	-	*SI Appendix*, Fig. S12
$I_{t=0}$	Initial proportion of infected birds	0.1	-	*SI Appendix*, Fig. S12
$R_{t=0}$	Initial proportion of recovered birds	0.6	-	*SI Appendix*, Fig. S12

Table S9 Parameters to simulate transmission of LPAIV strains in the individual-based model.

Parameter	Explaination	Value	Unit	Source
β	Transmission rate parameter	1.433×10^{-5}	day^{-1}	-
ε	Virus decaying rate in environment	0.2 (0.02-0.63)	-	(23)
γ	Recovery rate from the infection	1/7	day^{-1}	(24)
$1/\gamma$	Max infection duration	7	*day*	(24)
ϕ	Contribution of coinfection to $LPAIV_{poultry}$ transmission	0.5	-	-
ρ	Strength of partial immunity from previous infection	0.9 (0 - 0.9)	-	(4, 5)
σ	Cross-species transmission rate	4.8×10^{-5}	-	(3)
τ	Reassortment efficiency	1.9×10^{-3} (8.9×10^{-6} - 4.8×10^{-2})	$year^{-1}$	(6)

Table S10 Accuracy of the simulated spatial distribution of wild birds during fall migration in our individual-based model. The dBBMM presence and dBBMM absence indicate the sites identified by the dynamic Brownian Bridge Movement Model with telemetry tracking data, and the IBM presence and IBM absence indicate the sites used by Greater White-fronted geese in the individual-based model simulations.

	IBM presence	IBM absence	Sum
dBBMM presence	36	5	41
dBBMM absence	7	8	15
Sum	43	13	56

SI References

1. S. Yin, *et al.*, Habitat loss exacerbates pathogen spread: An Agent-based model of avian influenza infection in migratory waterfowl. *PLoS Comput Biol* **18**, e1009577 (2022).

2. S. Yin, *et al.*, Effects of migration network configuration and migration synchrony on infection prevalence in geese. *J Theor Biol* **502**, 110315 (2020).

3. H. Ren, *et al.*, Ecological dynamics of influenza A viruses: cross-species transmission and global migration. *Sci Rep* **6**, 36839 (2016).

4. J. G. B. van Dijk, *et al.*, A comparative study of the innate humoral immune response to avian influenza virus in wild and domestic mallards. *Front Microbiol* **11** (2020).

5. N. Latorre-Margalef, *et al.*, Competition between influenza A virus subtypes through heterosubtypic immunity modulates re-infection and antibody dynamics in the mallard duck. *PLoS Pathog* **13**, e1006419 (2017).

6. L. Lu, S. J. Lycett, A. J. Leigh Brown, Reassortment patterns of avian influenza virus internal segments among different subtypes. *BMC Evolutionary Biology* **14**, 16 (2014).

7. X. Wang, *et al.*, Gainers and losers of surface and terrestrial water resources in China during 1989–2016. *Nat Commun* **11**, 3471 (2020).

8. Copernicus Climate Change Service, Climate Data Store, Land cover classification gridded maps from 1992 to present derived from satellite observation. https://doi.org/10.24381/cds.006f2c9a. Deposited 2019.

9. D. Mao, *et al.*, National wetland mapping in China: A new product resulting from object-based and hierarchical classification of Landsat 8 OLI images. *ISPRS Journal of Photogrammetry and Remote Sensing* **164**, 11–25 (2020).

10. G. Zhang, *et al.*, Spatiotemporal patterns of paddy rice croplands in China and India from 2000 to 2015. *Sci Total Environ* **579**, 82–92 (2017).

11. Center for International Earth Science Information Network - CIESIN - Columbia University, Gridded Population of the World, Version 4 (GPWv4): Population Density, Revision 11 (Version 4.11). NASA Socioeconomic Data and Applications Center (SEDAC). https://doi.org/10.7927/H49C6VHW. Deposited 2017.

12. J. R. Meijer, M. A. J. Huijbregts, K. C. G. J. Schotten, A. M. Schipper, Global patterns of current and future road infrastructure. *Environmental Research Letters* **13**, 64006 (2018).

13. M. Gilbert, *et al.*, Global distribution data for cattle, buffaloes, horses, sheep, goats, pigs, chickens and ducks in 2010. *Sci Data* **5**, 180227 (2018).

14. B. L. Sullivan, *et al.*, eBird: A citizen-based bird observation network in the biological sciences. *Biol Conserv* **142**, 2282–2292 (2009).

15. Y. Si, *et al.*, Spring migration patterns, habitat use, and stopover site protection status for two declining waterfowl species wintering in China as revealed by satellite tracking. *Ecol Evol* **8**, 6280–6289 (2018).

16. P. Rohani, R. Breban, D. E. Stallknecht, J. M. Drake, Environmental transmission of low pathogenicity avian influenza viruses and its implications for pathogen invasion. *Proc. Natl. Acad. Sci. U.S.A.* **106**, 10365–10369 (2009).

17. X. Deng, *et al.*, Spring migration duration exceeds that of autumn migration in Far East Asian Greater White-fronted geese (Anser albifrons). *Avian Res* **10**, 1–11 (2019).

18. Y. Xu, *et al.*, A network approach to prioritize conservation efforts for migratory birds. *Conserv Biol* **34**, 416–426 (2020).

19. J. B. Dunning Jr., *CRC Handbook of Avian Body Masses*, J. B. Dunning Jr., Ed., 2nd Ed. (CRC Press, 2008).

20. A. D. Fox, “The Greenland White-fronted goose Anser albifrons flavirostris: The annual cycle of a migratory herbivore on the European continental fringe,” National Environmental Research Institute, Denmark. (2003).

21. I. Newton, Migration mortality in birds. *Ibis* **2024** (2024).

22. D. Sun, *et al.*, The impact of different relative humidity levels on the production performance, slaughter performance, and meat quality of white pekin ducks aged 4 to 42 days. *Anim* **13**, 3711 (2023).

23. V. L. Brown, P. Rohani, The consequences of climate change at an avian influenza “hotspot.” *BIOLOGY LETTERS* **8**, 1036–1039 (2012).

24. V. Hénaux, M. D. Samuel, Avian influenza shedding patterns in waterfowl: implications for surveillance, environmental transmission, and disease spread. *J Wildl Dis* **47**, 566–578 (2011).
